# Dual-Function Biomaterials for Postoperative Osteosarcoma: Tumor Suppression and Bone Regeneration

**DOI:** 10.34133/research.0978

**Published:** 2025-11-17

**Authors:** Hui Dong, Qing-Yi Zhang, Zi-Yuan Feng, Kai Huang, Rong Nie, Lu Li, Hong Duan, Hui-Qi Xie

**Affiliations:** ^1^ Department of Orthopedic Surgery and Orthopedic Research Institute, Stem Cell and Tissue Engineering Research Center, State Key Laboratory of Biotherapy, West China Hospital, Sichuan University, Chengdu 610041, Sichuan, China.; ^2^ Department of Medical Oncology, Cancer Center, West China Hospital, Sichuan University, Chengdu 610041, Sichuan, China.; ^3^ Lung Cancer Center, West China Hospital, Sichuan University, Chengdu 610041, Sichuan, China.

## Abstract

Osteosarcoma, a primary malignant bone tumor originating from mesenchymal cells, is the most prevalent bone malignancy in children and adolescents. Current standard treatment involves aggressive surgical resection aimed at maximal tumor removal; however, this inevitably creates extensive bone defects that substantially impair patients’ quality of life. Furthermore, the risks of postoperative tumor recurrence and metastasis critically influence therapeutic outcomes. With the continuous advancement of biomaterials, their applications in postsurgical osteosarcoma management have increased substantially. Biomaterial-based strategies are enabling diverse antineoplastic function and bone regeneration strategies. However, previous biomaterial strategies have typically focused on osteogenic or antitumor functions in isolation. The current research frontier is shifting toward developing integrated strategies that simultaneously achieve both effective tumor suppression and functional bone regeneration. This review strategically categorizes these emerging strategies into 3 distinct approaches: (a) Traditional bifunctional strategies: Integrating coloaded osteogenic and antitumor agents within a carrier. (b) Enhanced antitumor bifunctional strategies: Incorporating components or designs that actively boost the potency of the antitumor modality beyond simple codelivery. (c) Temporally controlled sequential strategies: Engineered to perform antitumor and pro-regeneration functions in a defined, sequential order. By critically analyzing the cutting-edge biomaterials employed in these strategies, we assessed their potential for clinical translation, emphasized the ongoing technical barriers, and outlined the challenges for future development.

## Introduction

Osteosarcoma is the most common primary bone tumor, characterized by rapid growth through the formation of tumor osteoid tissue and bone, either directly or indirectly via a cartilaginous stage. It predominantly affects children and adolescents, with an annual incidence of 4.7 cases per million in individuals aged 0 to 19 years, accounting for 8.9% of cancer-related deaths in this age group [1]. Although osteosarcoma can develop in any bone, it most frequently occurs around the knee joint and proximal humerus [2]. The primary clinical treatment for osteosarcoma involves surgical resection combined with adjuvant chemotherapy (CMT), which includes preoperative CMT, surgical tumor excision, and postoperative CMT, achieving a 5-year survival rate of 60% to 70% [3]. However, achieving complete surgical resection remains challenging due to the presence of microscopic satellite lesions, indistinct tumor margins, and the limitations of intraoperative frozen section analysis in hard surrounding tissues [4]. Residual tumor cells may remain in adjacent bone regions or at the resection margins, leading to rapid proliferation, local recurrence of the osteosarcoma, or metastasis. The 5-year survival rate for patients with metastatic or recurrent osteosarcoma is only 20% [5].

Current first-line CMT agents used in post-osteosarcoma surgery, such as doxorubicin (DOX), cisplatin (CDDP), gemcitabine (GEM), and methotrexate (MTX), are predominantly administered systemically. However, poor intramedullary blood flow and the blood–bone marrow barrier keep tumor-site drug concentrations low, while systemically administered agents are rapidly metabolized by the liver and kidneys; the resulting short half-life and low bioavailability blunt their therapeutic impact [6,7]. Furthermore, this approach results in nonselective cytotoxicity and adverse effects on healthy tissues, including cardiomyopathy, vomiting, and alopecia [6,8]. Turning to radiotherapy (RDT), most osteosarcomas have low radiosensitivity; thus, RDT is relatively ineffective for tumor control. Even with high-dose irradiation, many patients still have tumor remnants. Combined with the risk of normal tissue damage, RDT’s clinical use is limited to palliative or adjuvant treatment. In recent years, immunotherapy (IMT) for osteosarcoma has garnered increasing attention. The immune checkpoint molecule programmed cell death protein 1 (PD-1) is highly expressed in osteosarcoma. It exerts its effect by binding to programmed cell death ligand 1 (PD-L1) on the surface of T cells—this interaction inhibits T cell activation and proliferation, and ultimately impairs T cells’ cytotoxic capacity against tumor cells [9]. For this reason, PD-1/PD-L1 inhibitors are regarded as promising therapeutic agents for osteosarcoma. However, the response rates in phase II clinical trials of PD-1 monoclonal antibodies in osteosarcoma patients have been disappointing [10]. To address these therapeutic challenges, the application of biomaterials in osteosarcoma treatment has achieved remarkable progress. For example, they enable localized delivery of antineoplastic drugs, checkpoint inhibitors, and radioactive substances. This not only increases drug concentrations at the tumor site but also reduces systemic toxicity. Additionally, a number of novel antitumor therapeutic modalities have emerged. Traditional hyperthermia lacks selectivity between target and surrounding healthy tissues, often leading to burns in normal tissue [11]. Biomaterials enhance hyperthermia therapy by providing better targeting and precise temperature control. In brief, these methods induce heat generation in the materials upon exposure to external light sources, microwaves, or electromagnetic fields, thereby effectively destroying tumor cells. Photothermal therapy (PTT) materials primarily absorb light in the near-infrared (NIR) region [12–15], which limits their tissue penetration depth. Magnetic hyperthermia therapy (MHT) and microwave thermal therapy (MTT) offer deeper tissue penetration, enabling effective treatment of tumors located within deeper bone structures [16,17]. Chemodynamic therapy (CDT) leverages the acidic, H_2_O_2_-rich tumor microenvironment to catalyze Fenton or Fenton-like reactions by biomaterials, spawning reactive oxygen species (ROS) to eradicate cancer cells [18]. Photodynamic therapy (PDT) and sonodynamic therapy (SDT) generate products similar to those of CDT, triggered by light and ultrasound, respectively, all aiming to induce tumor cell death via ROS production [19–21].

For patients, osteosarcoma resection often leaves critical bone defects that impair mobility and quality of life, especially in adolescents who are still growing and highly physically active. Therefore, in addition to preventing recurrence, restoring bone structure and function is a central objective of postoperative management. Bone regeneration is a complex process involving the recruitment, proliferation, and osteogenic differentiation of bone marrow stem cells (BMSCs), as well as the regeneration and ingrowth of blood vessels and nerves and immune modulation, among other processes [22–29]. Autologous bone is the gold standard for bone repair, but the quantity of donor bone available in patients is limited, and harvesting it can lead to various complications [30,31]. Allogeneic bone, as a substitute, can address the issue of limited bone availability but carries risks of disease transmission, immune response, and bone resorption [32,33]. In light of these challenges, various biomaterials have been developed for clinical bone tissue repair that are not only used as structural scaffolds but also increasingly designed to actively participate in the healing process [34]. Metallic biomaterials are among the first to be utilized for the repair of bone defects due to their superior mechanical properties and biocompatibility [35]. Many metal ions, such as Mg and Zn, not only can drive osteogenic differentiation but also can synergistically enhance vascularization via endothelial activation, while initiating neurogenesis at the implantation site [27,36–38]. Inorganic nonmetallic materials, primarily including calcium phosphate ceramics and bioactive glass (BG), inherently possess excellent osteoconductive and osteoinductive properties [39,40]. The release of their degradation products exerts a promoting effect on osteoblast differentiation and extracellular matrix (ECM) formation. Organic materials are categorized into natural polymers and synthetic organic materials, where natural polymers possess superior biocompatibility and biomimetic structures, while synthetic polymers exhibit distinct advantages in terms of mechanical properties and processability [41]. Combinations of multiple materials form hydrogels, scaffolds, coatings, and films, providing an optimal environment for osteoblast adhesion and proliferation while also serving as delivery systems for the targeted release of osteogenic drugs, cytokines, or mesenchymal stem cells [42,43]. However, the pursuit of complete tumor eradication often directly opposes the conditions required for effective bone regeneration. CMT, IMT, and other adjuvant treatments, while essential for preventing recurrence, can simultaneously inhibit osteoblast activity and angiogenesis, thereby impeding bone repair. Conversely, the growth factors and cell-friendly microenvironments that promote regeneration may inadvertently support the survival of residual tumor cells. Against this critical backdrop, there is an urgent need for biomaterials to evolve into versatile strategies that can simultaneously meet this dual requirement: effectively supporting bone regeneration while mitigating the risk of tumor recurrence.

**Fig. 1.**
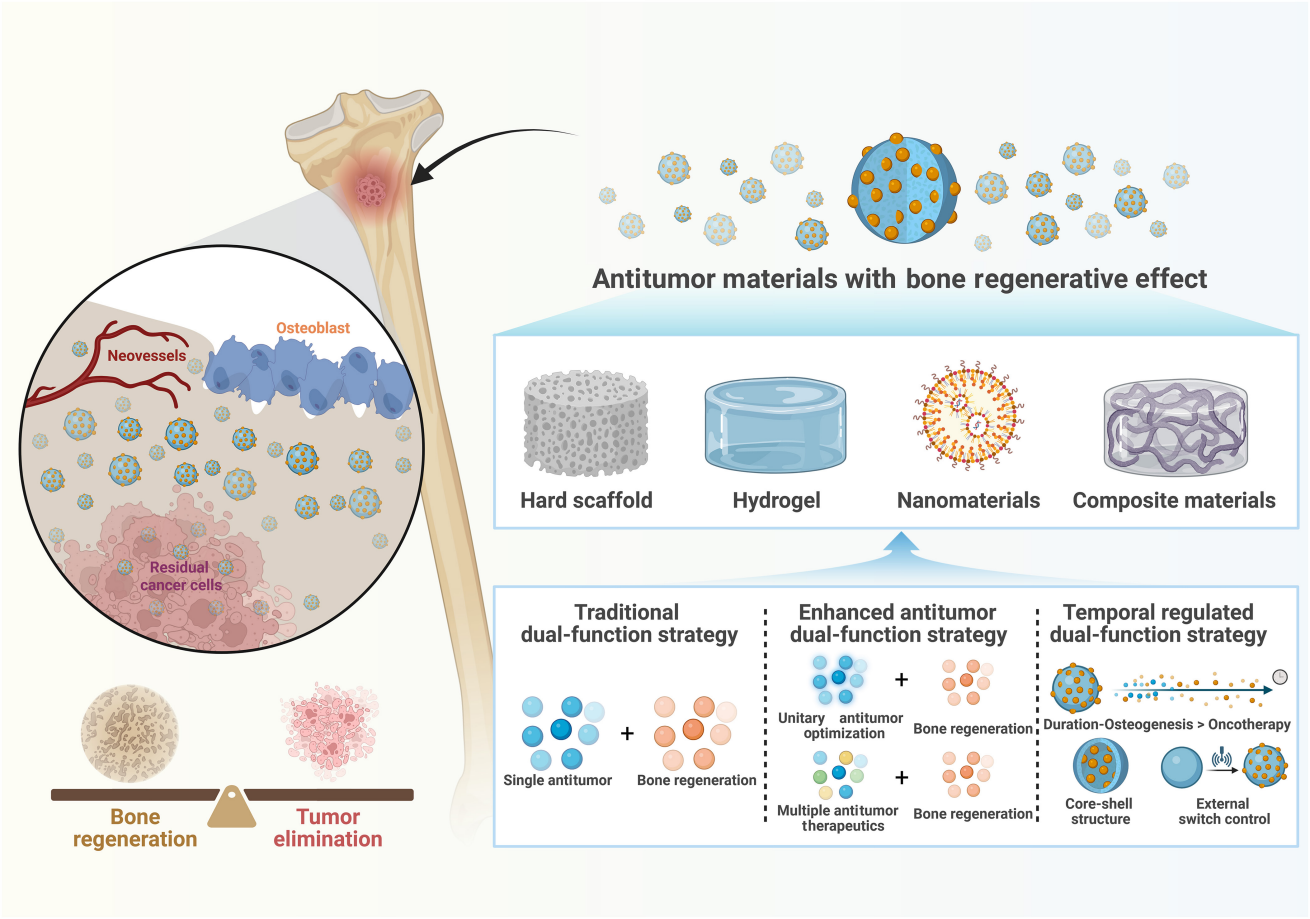
Biomaterial-based bifunctional strategies for antitumor and bone regeneration.

**Fig. 2.**
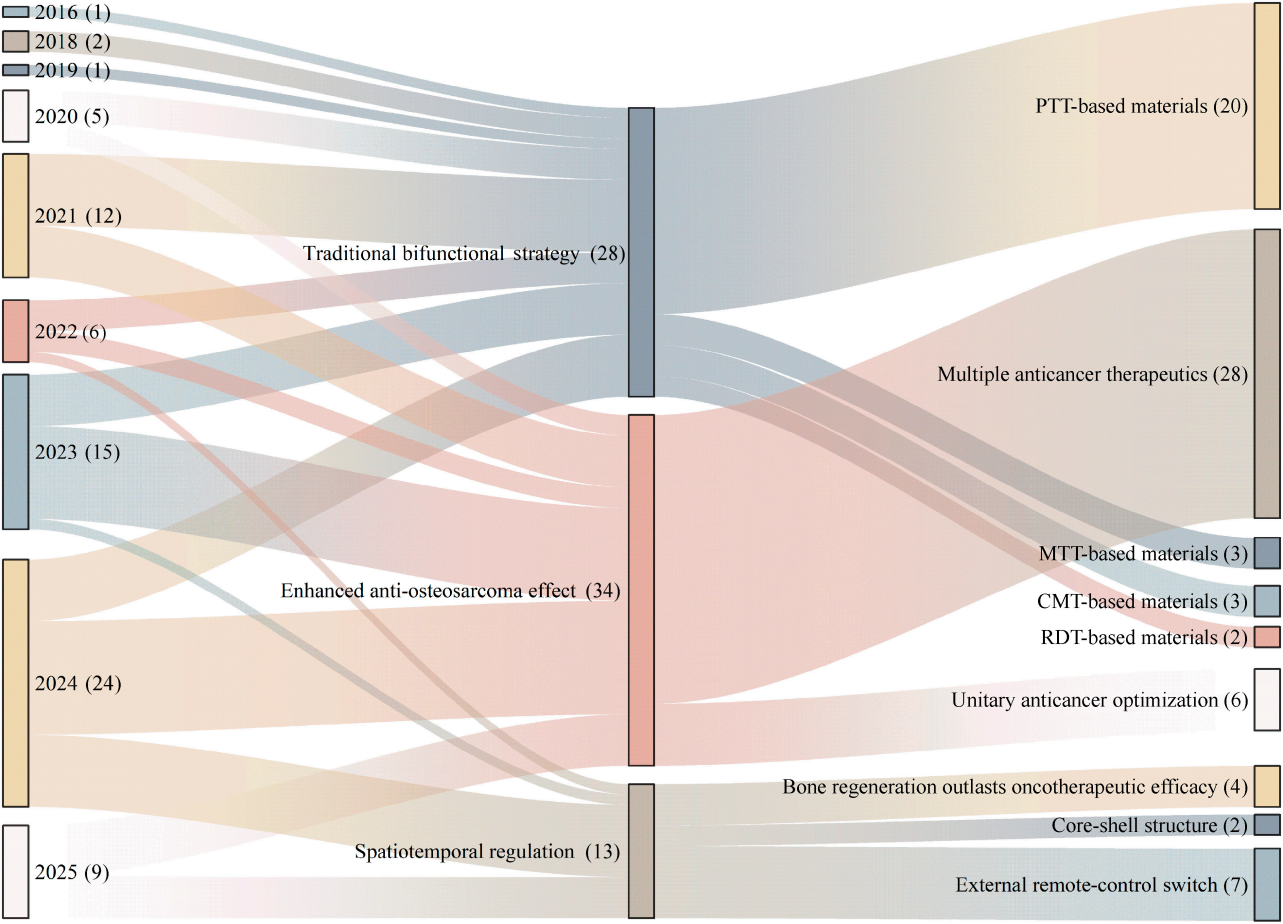
Sankey diagram-based bibliometric analysis of bifunctional strategic materials (2015 to present).

Currently, bifunctional biomaterials for osteosarcoma treatment mainly focus on nanomaterials, hydrogels, and hard scaffolds. Nanomaterials, defined as materials and structures with dimensions ranging from 1 to 100 nm, can be specifically designed or precisely controlled, possessing controllable surface properties, specific surface area, mechanical properties, and optical properties [44]. Common types of nanomaterials include nanoparticles, nanofilms, nanocomposites, and nanoporous materials, which have been widely applied in both osteosarcoma treatment and bone regeneration [45,46]. Hydrogels are 3-dimensional (3D) porous network gels with excellent biocompatibility that can mimic the ECM. They are structurally degradable and highly adaptable in shape, making them suitable for fitting irregular bone defects. Additionally, hydrogels have strong drug-loading capabilities, allowing them to serve as carriers for antitumor and osteogenic components [47,48]. Advancements in technologies such as 3D printing, coating techniques, and electrospinning have substantially enhanced the flexibility in assembling hard scaffolds, providing more diverse assembly strategies. Notably, 3D printing technology enables precise control over the shape of implants according to the anatomical structure of post-tumor resection defects, thereby achieving personalized customization to meet the needs of post-resection repair. 3D-printed prostheses have been widely used clinically to match the irregular shapes of bone defects resulting from tumor surgery [49]. However, stand-alone nanomaterials often struggle to conform to irregular bone defects, and hydrogels typically exhibit poor mechanical strength. Hard scaffolds, while generally more mechanically robust, often lack the biocompatibility of hydrogels. Therefore, these materials are often combined to leverage their complementary properties. This review systematically synthesizes the latest research advances in the development of such bifunctional biomaterials. For the first time, it innovatively proposes a tripartite classification system grounded in the core characteristics of bifunctional strategies, specifically encompassing (a) traditional bifunctional strategies, (b) enhanced antitumor strategies, and (c) temporal regulation strategies (Fig. 1). Building on this framework, the review further delineates the developmental trajectory of these materials—from the design of traditional simple composite structures to the evolution of complex temporal control systems (Fig. 2). Furthermore, this paper focuses on preliminary discussions regarding the key pain points in the clinical translation of these materials—such as biosafety and infection risks—and provides preliminary insights into future research directions, in the hope of offering some theoretical references for the subsequent advancement of this field.

## Traditional Bifunctional Strategies

In the present research context, we offer a formal classification and definition of traditional bifunctional therapeutic strategies. These are constructs that are systematically integrated, combining a single antitumor component with bone regeneration element (Table 1).

**Table 1.** Traditional bifunctional strategic materials

Strategy	Biological materials	Antitumor	Osteogenesis	Specific details	Ref.
PTT-based materials	Pluronic F127/BG/BPQDs	BP (808 nm)	BPQDs/BG	Pluronic F127 encapsulates BP in BG-modified micellar cores, triggering microwave-driven BPNSs→BPQDs conversion.	[52]
	NAGA/GelMA/Gd/MOS_2_	MOS_2_ (808 nm, 26.26%)	Gd	NAGA/GelMA hydrogel incorporated with MOS_2_ and Gd. Gd allows additional MRI.	[70]
	CMP/PAM/MgO/PDAM	PDAM (808 nm)	MgO	CMP/PAM hydrogel incorporated with MgO and PDAM nanoparticles.	[53]
	PEEK/MOS_2_/nHA	MOS_2_ (808 nm)	nHA	PEEK scaffold incorporated with MOS_2_ and nHA.	[55]
	β-TCP/Cu-TCPP	Cu-TCPP (808 nm)	Cu/β-TCP	β-TCP scaffold incorporated with Cu-TCPP.	[54]
	BP/BG	BP (808 nm)	BP/BG	BG scaffold incorporated with BP.	[56]
	CS/nHA/CD	CD (808 nm)	CS/nHA/CD	CS scaffold incorporated with nHA and CD.	[57]
	PCL/SrCuS_i4_O_10_	SrCuS_i4_O_10_ (1,064 nm, 46.3%)	Sr/Cu/SI	PCL scaffold incorporated with SrCuS_i4_O_10_.	[58]
	Ti_3_C_2_ MXene/BG	Ti_3_C_2_ MXene (808 nm)	Ti_3_C_2_ MXene/BG	BG scaffold incorporated with Ti_3_C_2_ MXene.	[59]
	Nb (2)C MXene/BG	Nb (2)C MXene (1,064 nm)	Nb (2)C MXene/BG	BG scaffold incorporated with Nb (2)C MXene.	[60]
	CaCO_3_/PCL/CaCuSi_4_O_10_	SrCuSi_4_O_10_ (1,064 nm)	CaCO_3_/Ca SrCuSi_4_O_10_	PCL scaffold with CaCO_3_ and CaCSrCuSi_4_O_10_ loaded surface coating.	[61]
	PLGA/Mg	Mg (808 nm)	Mg	Porous PLGA scaffold incorporated with Mg.	[62]
	BG/GNP	GNP (808 nm)	BG	BGNF crosslinked by GNP-Gel into a stable 3D network.	[63]
	BG/bismuth	Bismuth (808 nm)	BG	BG scaffold with bismuth loaded surface coating.	[64]
	β-TCP/MoS_2_/PDA/BMP2/IGF-1	MoS_2_/PDA (808 nm)	BMP2/IGF-1	β-TCP scaffold with MoS_2_, PDA, BMP2, and IGF-1 loaded surface coating.	[65]
	β-TCP/carbon aerogel	Carbon aerogel (808 nm)	β-TCP/carbon aerogel	β-TCP scaffold with carbon aerogel loaded surface coating.	[66]
	Ti_6_Al_4_V/TiO_2_	TiO_2_ (808 nm)	TiO_2_	Ti_6_Al_4_V scaffold with TiO_2_ loaded surface coating.	[67]
	Ti_6_Al_4_V/TiO_2_/TiP/Gel/HA/Alendronate	TiO_2_ (808 nm)	TiO_2_/HA/Alendronate	Ti_6_Al_4_V scaffold with TiO_2_, TiP, Gel, HA, and alendronate loaded surface coating.	[68]
	Nd/Mn/whitlockite/SA	Nd (808 nm)	Nd/Mn/whitlockite	Nd10%Mn10%WH-SA hydrogel formed via SA-Ca^2+^ electrostatic and H-bond crosslinking. Nd10% Mn10% WHSA can additionally monitor the temperature.	[72]
	MB/CMB	CMB (1,064, 29.50%)	MB/CMB	BG scaffold incorporated with CMB.CMB additionally has ROS scavenging capacity.	[78]
MHT-based materials	MeCFO/GelMA	CFO	MeCFO	GelMA hydrogel incorporated with MeCFO.	[80]
	SPION/HA/CS/PVA	SPION	HA/CS	HA/CS hydrogel incorporated with SPION.	[81]
	Fe_3_O_4_/Goβ-TCP	Fe_3_O_4_	Go/β-TCP	β-TCP scaffold with Go and Fe_3_O_4_ loaded surface coating.	[82]
CMT-based materials	HEMA/MMA/DOX	DOX	HEMA/MMA	HEMA/MMA scaffold with DOX loaded surface coating.	[83]
	Sr/CaPo_4_/MTX/DOX	MTX/DOX	Sr/CaPo_4_	CaPo4 scaffold incorporated with Sr, MTX and DOX.	[84]
	PLGA-PEG-PLGA/Ti_6_Al_4_V simvastatin	Simvastatin	Simvastatin	Ti_6_Al_4_V scaffold with simvastatin loaded PLGA-PEG-PLGA hydrogel coating.	[87]
RDT-based materials	Poloxamer 407 hydrogel/BG/holmium	BG/holmium	BG	Poloxamer 407 hydrogel incorporated BG loaded holmium.	[88]
	CB/SA/gelatin	CB	SA/gelatin	SA/gelatin hydrogel incorporated CB.	[89]

### PTT-based traditional bifunctional strategic materials

**Fig. 3.**
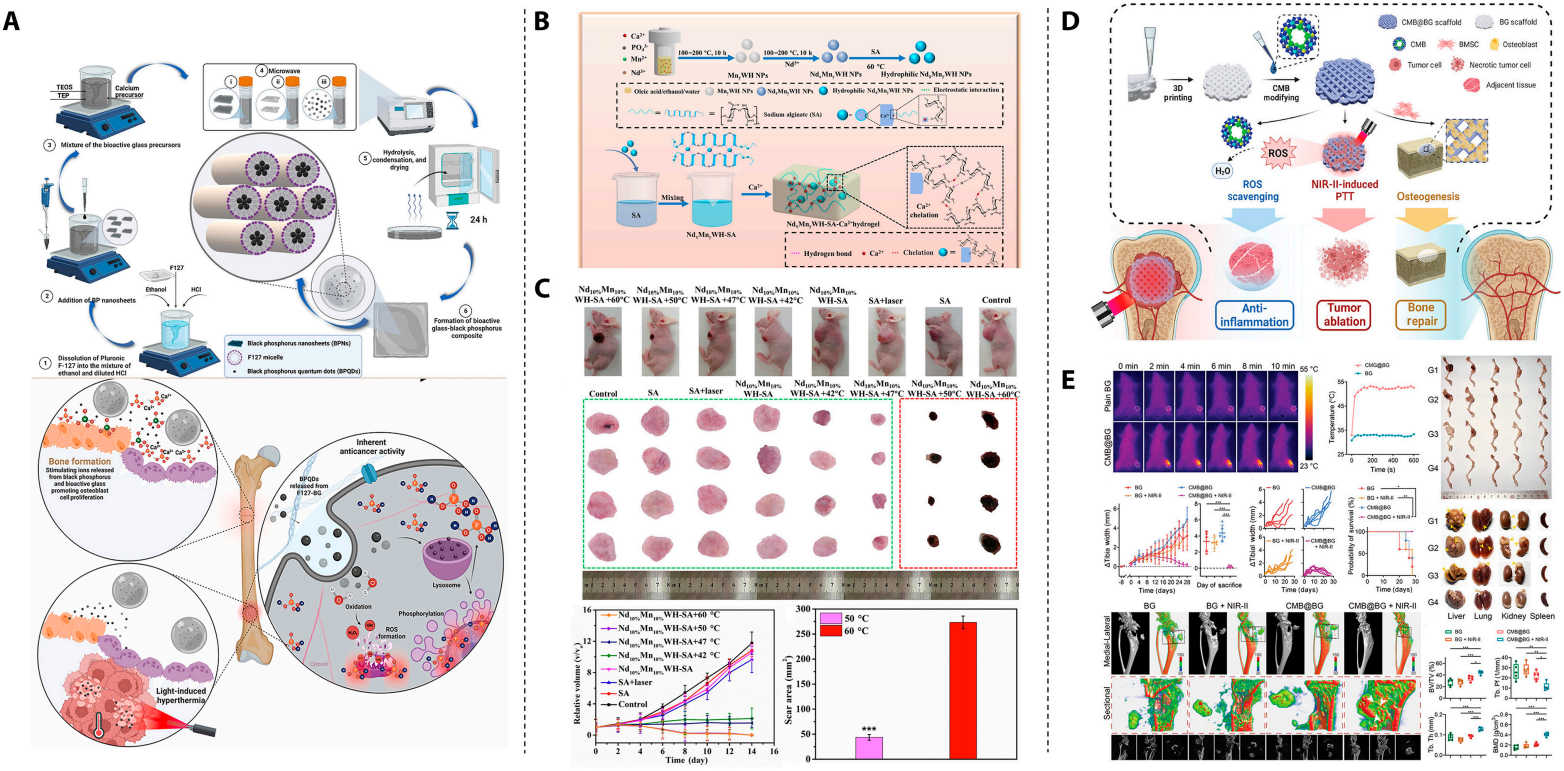
PTT-based traditional bifunctional strategic materials. (A) Synthetic procedure for the preparation of F127-BG-BPQDs for bone cancer therapy and regeneration. Reproduced with permission [52]. Copyright 2024, Elsevier. (B) The mechanism of preparing Nd*_x_*Mn*_y_*WH-SA, followed by their application. (C) Photothermal antitumor effects of the composite hydrogel at different temperatures in vivo. Reproduced with permission [72]. Copyright 2024, Elsevier. (D) Schematic illustration of CMB@BG scaffolds equipped with self-assembling CMB nanowheel crystals with photothermal catalytic effects as a 3-in-1 solution for osteosarcoma. (E) The 3-in-1 therapeutic effect of CMB@BG scaffolds in an orthotopic osteosarcoma animal model. Reproduced with permission [78]. Copyright 2024, John Wiley and Sons.

PTT has emerged as a pivotal modality for osteosarcoma treatment, where advanced biomaterials address dual imperatives: effective tumor ablation and osteogenic microenvironment orchestration. Common photothermal materials include noble metal nanoparticles, carbon nanotubes, black phosphorus (BP), MXenes, sulfides, polydopamine (PDA), indocyanine green, Prussian blue, and polypyrrole (Ppy), among others [50]. A fundamental challenge in their application lies in the inherent contradiction between biodegradability and stability. Biodegradability demands that the material gradually decomposes and is cleared in the biological environment to avoid long-term accumulation, while stability requires it to maintain structural integrity and photothermal conversion efficiency throughout the therapeutic cycle. A prime example is BP, which stands out due to its intrinsic osteogenic by-products but suffers from rapid oxidative degradation in internal environments, hindering its clinical translation [51,52]. To address the stability issue of photothermal materials in degrading biological environments, a key strategy has been developed: physical encapsulation and confinement, which works by isolating photothermal materials from the degrading environment. For example, Bigham et al. [52] synthesized a BG (BG-BP) composite material via evaporation-induced self-assembly (Fig. 3A). Using the amphiphilic nonionic surfactant Pluronic F127 as a protective agent during the drying process, BP was encapsulated within the hydrophobic core of micelles, followed by the formation of the BG component around the core. Subsequent irradiation transformed the composite into BP quantum dots (BPQDs). The micelles not only shielded BP during scaffold synthesis but also confined the BP fragments into quantum dots upon NIR irradiation, whose small size further slowed oxidative degradation. Similarly, hydrogels can act as protective matrices for photothermal nanomaterials. Li et al. [53] developed bioactive nanoparticles comprising magnesium oxide nanoparticle cores and poly(2-aminoethyl methacrylate)-grafted PDA (PDAM-2AM) shells. These nanoparticles were subsequently incorporated into a pre-gel system composed of phosphonate-modified meth acrylamide chitosan (CMP) and polyacrylamide (PAM). The resultant 5NP/CMP@PAM hydrogel demonstrated complete suppression of osteosarcoma recurrence both in vitro and in vivo when activated by NIR laser irradiation. Furthermore, in critical-sized rat calva rial defect models, the hydrogel exhibited superior bone regeneration capabilities, demonstrating its dual functionality in oncological therapy and bone tissue engineering. Incorporating or physically impregnating photothermal-responsive nanomaterials directly into the bone scaffold fabrication process provides a convenient method for stabilizing photothermal materials and creating a dual-functional platform [54–61]. Long et al. [62] used low-temperature rapid prototyping to 3D print porous poly lactic-co-glycolic acid (PLGA) scaffolds doped with Mg particles, thus the ability of composite scaffolds to obtain the photothermal effect of magnesium particles and promote bone regeneration. Wang et al. [63] combined flexible BG nanofibers and genipin-crosslinked gelatin (GNP) to fabricate porous 3D scaffolds via ice-templating and freeze-drying techniques. The resulting scaffolds exhibited a nanofibrous structure, closely mimicking the ECM. The GNP gel acted as a photothermal agent, endowing the GNP gel/BGNF 3D scaffolds with potent photothermal antitumor and antibacterial properties. Creating photothermal coatings directly on bone scaffold surfaces presents another convenient strategy. Examples include bismuth coatings on BG scaffolds [64], molybdenum disulfide (MoS_2_)/PDA or carbon aerogel coatings on β-tricalcium phosphate (β-TCP) scaffolds [65,66], and TiO_2_ (H-TiO_2_) coatings on 3D-printed titanium scaffolds [67,68].

The evolution of dual-functional strategies has underscored the rising importance of theranostic materials, which are designed for both treatment and diagnostic monitoring, thus expanding their functional capabilities. N-acryloyl glycinamide (NAGA) possesses desirable mechanical properties suitable for providing mechanical support during bone regeneration; however, its nondegradable nature limits its application. Gadolinium (Gd), exhibiting osteogenic activity, also functions as a highly effective contrast agent for magnetic resonance imaging (MRI) [69]. Huang et al. [70] developed a multifunctional hydrogel comprising Gd and MoS_2_ codoped NAGA/gelatin methacryloyl (GelMA). The addition of GelMA makes this hydrogel exhibit favorable mechanical properties and controllable degradation. This hydrogel possessed excellent photothermal capabilities due to MoS2, enabling tumor cell ablation and bacterial growth inhibition both in vitro and in vivo. The Gd complex enabled MRI monitoring of hydrogel location and degradation. Furthermore, the gradual release of Gd^3+^ during GMNG hydrogel degradation promoted osteogenesis in vivo. PTT based on conventional photothermal agents suffers from the inability to monitor in situ temperature. This limitation can lead to overheating, causing damage to surrounding healthy tissues, or insufficient heating, resulting in incomplete tumor ablation. Neodymium (Nd) ions, belonging to the rare-earth family, exhibit photothermal properties and fluorescence upon 808-nm laser excitation [71]. Heng et al. [72] synthesized composite nanoparticles by incorporating Nd and manganese (Mn) ions into whitlockite nanoparticles (a calcium and phosphate-containing inorganic mineral). These nanoparticles were then conjugated with sodium alginate (SA) via hydrogen bonding to form Nd10%Mn10%WH-SA (Fig. 3B). Upon implantation of Nd10%Mn10%WH-SA into the tumor site, the PTT temperature could be controlled by monitoring changes in fluorescence intensity. In vivo experiments demonstrated that optimal antitumor efficacy was achieved at 50 °C without causing substantial skin burns (Fig. 3C). Moreover, the presence of Mn and whitlockite contributed to substantial bone regeneration in a rabbit femoral defect model.

With more in-depth research into PTT, a groundbreaking theory has emerged suggesting that PTT, which utilizes near-infrared lasers to induce heat treatment, could be a double-edged sword. While it effectively eliminates tumor cells, it can also trigger adverse inflammatory responses [73,74]. Although it is generally believed that ROS can eliminate tumor cells, it has also been suggested that ROS production causes a local inflammatory microenvironment that promotes tumor recurrence and should be removed [75,76]. Polyoxometalates, a unique class of molecular clusters composed of one or more transition metals and oxygen atoms, exhibit enhanced NIR absorption properties [77]. Lu et al. [78] doped molybdenum blue (MB) nanoclusters, which possess a well-defined cyclic structure, with cerium ions, forming a mixed-valence state of Mo^6+^/^5+^ and stable Ce^3+^. The substitution of Ce^3+^ enhanced SPR performance, thereby augmenting the capability of PTT-induced tumor ablation. The multivalent states of Mo endowed it with ROS scavenging ability, while the Ce^3+^ substitution led to an increase in the adsorption energy of the intermediates, consequently enhancing the ROS scavenging capability. This modification preserved the symmetrical ring structure and photothermal effect while imparting ROS scavenging ability to it. The resulting Ce-doped MB nanoclusters were loaded onto 3D-printed BG (CMB@BG) scaffolds, creating a novel trimodal therapeutic approach for osteosarcoma, combining antitumor, anti-inflammatory, and bone regenerative properties (Fig. 3D and E).

### Non-PTT-based traditional bifunctional strategic materials

#### MHT-based traditional bifunctional strategic materials

**Fig. 4.**
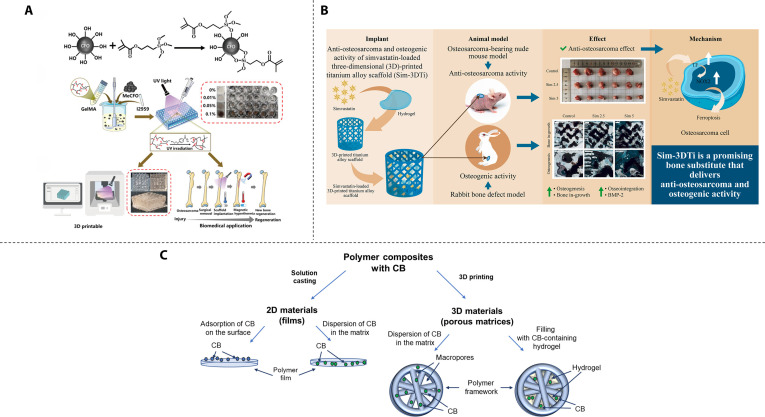
Non-PTT-based traditional bifunctional strategic materials. (A) Schematic diagram of synthetic routes and application of MeCFO/GelMA hydrogels. Reproduced with permission [80]. Copyright 2023, Frontiers. (B) Schematic diagram of synthetic routes, application, and mechanism of composite hydrogels. Reproduced with permission [87]. Copyright 2024, Elsevier. (C) Design of the composite materials considered in the study. Reproduced with permission [89]. Copyright 2022, MDPI.

While dual-functional strategies based on PTT have brought much promise and are flourishing, they are limited by the shallow tissue penetration depth. MHT, which leverages the deep tissue penetration of alternating magnetic fields, can overcome this limitation [79]. Shi et al. [80] grafted methacrylate groups onto the surface of cobalt ferrite (CoFe_2_O_4_ [CFO]) magnetic nanoparticles to reduce cytotoxicity and improve biocompatibility. These modified CFO nanoparticles (MeCFO) were then incorporated into a GelMA hydrogel, resulting in a high-performance magnetic hydrogel (Fig. 4A). This hydrogel facilitated osteogenic differentiation of bone marrow mesenchymal stem cells while enabling magnetothermal therapy for the ablation of osteosarcoma cells. Tavares et al. [81] integrated hydroxyapatite (HA) particles into a polymer matrix composed of chitosan (CS) and polyvinyl alcohol (PVA). Superparamagnetic iron oxide nanoparticles (pSPIONs) were then added to the CS/PVA/HA paste at 3 different concentrations (1.92, 3.77, and 5.54 wt%) prior to scaffold fabrication. Scaffolds containing 3.77 and 5.54 wt% pSPIONs achieved temperature increases of 6.6 and 7.5 °C, respectively, during magnetothermal testing, demonstrating inhibitory effects on osteosarcoma. Furthermore, the presence of CS and HA promoted bone formation and integration. Similar to photothermal coatings, magnetothermal nanomaterials have also been employed as coatings on bone scaffolds. Zhang et al. [82] fabricated a 3D-printed β-TCP bioceramic scaffold coated with an Fe_3_O_4_ nanoparticle/graphene oxide (GO) nanocomposite layer. This coating conferred magnetothermal properties and enhanced osteogenesis to the β-TCP scaffold.

#### CMT-based traditional bifunctional strategic materials

Localized chemotherapeutic delivery systems represent a pivotal strategy for synchronizing osteosarcoma eradication and bone regeneration. These strategies overcome systemic limitations through targeted drug release. Sreeja et al. [83] fabricated layered porous scaffolds (HEMA/MMA) through free radical copolymerization of hydroxyethyl methacrylate (HEMA) and methyl methacrylate (MMA) and obtained an antitumor capability by immersing in DOX. The scaffold significantly promoted the osteogenic differentiation of rat BMSCs, while its special structure delayed the release of DOX for better tumor eradication. Lanzillotti et al. [84] synthesized injectable strontium-doped calcium phosphate self-hardening scaffolds, combining the osteoinductive capability of Sr and the well-recognized osteointegrative properties of calcium phosphate to maintain bone tissue regeneration. It could also serve as a drug delivery system, combining bone tissue regeneration and anticancer treatment by loading MTX and DOX. Beyond conventional chemotherapeutics, drug repurposing has yielded promising candidates with emerging antitumor efficacy. Simvastatin, a clinically established lipid-modulating agent, further exhibits osteogenic, antibacterial, and antitumor activities in preclinical studies; however, subtherapeutic concentrations at osseous and tumor sites due to first-pass metabolism markedly compromise its therapeutic efficacy for bone regeneration and oncological interventions [85,86]. Jing et al. [87] addressed this limitation by encapsulating simvastatin in thermosensitive PLGA-PEG-PLGA hydrogels and injecting the formulation into 3D titanium scaffolds for localized delivery, They demonstrated that it could effectively inhibit osteosarcoma and promote bone formation, and the anti-osteosarcoma mechanism was through up-regulating ferritin and nicotinamide adenine dinucleotide phosphate oxidase 2 levels to induce ferroptosis (Fig. 4B).

#### RDT-based traditional bifunctional strategic materials

Shifting the focus to radiotherapeutic modalities, biomaterial-mediated brachytherapy exhibits distinct advantages in terms of spatial control. Zambanini et al. [88] encapsulated holmium-containing BG particles in Poloxamer 407 hydrogel. Their study demonstrated that brachytherapy using this composite effectively induced osteosarcoma cell death. The optimal concentration of holmium was determined to be 5 wt.%, at which the highest efficacy was observed. Additionally, the BG promoted the proliferation of osteoprogenitor cells, further supporting bone regeneration. Stepanova et al. [89] encapsulated 3D-printed matrices of aliphatic polyesters containing cyclic borate (CB) within a composite hydrogel composed of SA and gelatin (Fig. 4C). This innovative approach proposes a novel strategy for integrating boron neutron capture therapy with bone regeneration, offering new possibilities for the treatment of bone-related malignancies.

Traditional bifunctional strategies, which centered on the direct codelivery of antitumor and osteogenic components, provided a foundational concept for postoperative osteosarcoma management. However, the therapeutic efficacy of this approach was ultimately limited by insufficient tumor eradication and an unresolved conflict in the timing between tumor elimination and bone regeneration.

## Bifunctional Strategies Based on Enhanced Antitumor Effect

Successful antitumor treatment post-osteosarcoma surgery is the most pivotal factor in patient prognosis. Therefore, researchers have been focusing on enhancing tumor eradication efficiency within the framework of dual-function strategies (Table 2).

**Table 2.** Bifunctional strategic materials based on enhanced anti-osteosarcoma effect

Strategy	Biological materials	Antitumor	Osteogenesis	Specific details	Ref.
Unitary anticancer optimization	PCL/nHA/MgO_2_/PDA	PDA—PTT MgO_2_—Increase photothermal sensitivity	nHA/MgO_2_	PC scaffold incorporated with nHA, MgO_2_, and PDA. MgO_2_ releases oxygen via hydrolysis, suppresses hypoxia-driven glycolysis and HSPs, enhancing tumor heat sensitivity.	[92]
	Fe_3_O_4_/MgCO_3_/GOx/PLGA	Fe_3_O_4_—MHT Fe_3_O_4_/GOx—Increase photothermal sensitivity	Mg	PLGA hydrogel incorporated Fe_3_O_4_, MgCO_3_, and Gox. GOx depletes glucose to block glycolysis/HSPs; Fe_3_O_4_ recycles H_2_O_2_ into O_2_, intensifying starvation therapy.	[95]
	SA-Cu-MXene/PLLA/BG	MXene—PTT SA-Cu—Increase photothermal sensitivity SA-Cu—CDT	Cu/MXene//BG	PLLA/BG hydrogel incorporated SA-Cu-MXene. SAzymes generate abundant ROS to disrupt HSPs’ structure and function, thereby enhancing tumor thermal sensitivity.	[97]
	BG/NS/IOX1	NS—PTT IOX1—Increase PTT sensitivity	BG	BG scaffold with NS loaded surface coating. IOX1 suppresses KDM3A to boost tumor epigenetic sensitivity to PTT.	[99]
	Se/Mg/Fe/LDH/BG	Fe—CDT Se—Increased CDT efficiency and sensitivity	Mg/Fe/BG	BG scaffold incorporated with MgFe-LDH/Se nanocomposite. SeNPs deplete GSH and elevate H_2_O_2_ levels, supplying precursors for enhanced CDT.	[105]
	Vanadium/BG/PLGA	Vanadium—CDT Vanadium—Increase CDT sensitivity	BG	BG/PLGA scaffold incorporated mixed-valence vanadium. Vanadium depletes GSH, enhancing CDT sensitivity.	[102]
Multiple anticancer therapeutics	BP/DOX/CS	BP—PTT DOX—CMT	BP	CS hydrogel incorporated with BP and DOX.	[109]
	GelMA/oxidized dextran/MMT-Sr/Ppy/DOX	Ppy—PTT DOX—CMT	MMT-Sr	GOMP hydrogel incorporated with Ppy, DOX, and MMT-Sr.	[110]
	GelAGE/Sr/PDA/DOX	PDA—PTT DOX—CMT	Sr	GelAGE hydrogel incorporated with PDA, DOX, and Sr.	[111]
	PEEK/Graphene/DDP/HA	Graphene—PTT DDP—CMT	HA	3D-printed PEEK scaffold with internal graphene and surface HA@DDP coating.	[113]
	HA/PCL/DOX/PDA	PDA—PTT DOX—CMT	nHA	PCL scaffold with HA/DOX-PDA loaded surface coating.	[114]
	DCM/PLGA/β-TCP/BP/DOX/P24	BP—PTT DOX—CMT	TCP/BP/P24	DCM/PLGA/β-TCP/BP scaffold incorporated with BP and DOX.	[115]
	CDHA/DOX/PDA	PDA—PTT DOX—CMT	CDHA	CDHA scaffold with DOX-PDA loaded surface coating.	[116]
	Whitlockite/Go/DOX	Go—PTT DOX—CMT	Go/Whitlockite	Whitlockite scaffold incorporated with Go and DOX.	[117]
	Se/Mg/nHA/PDA/Sericin/Oxidized chondroitin sulfate/CaO_2_	PDA—PTT Se—CMT	Mg/Ca/nHA	Hydrothermal Se/Mg-HAp nanorods, self-polymerized PDA-CaO_2_ nanospheres, and hydrazide-crosslinked Ser/OCS hydrogel.	[112]
	PDA/Pt (II)/PLLA/BG	PDA—PTT Pt (II)—CMT	BG	Pt (II) amidated to PDA, then laser-sintered into PLLA/BG.	[118]
	Au/CA	Au—PTT CA—CMT	CA	CA-conjugated Au nanorods via Au-catechol bonds	[121]
	MSN/PDA/Qr/Col	PDA—PTT Qr—CMT	Qr	Qr and PDA coloaded MSNs	[125]
	Methylcellulose/PLGA/CM/IR820	IR820—PTT CM—CMT	CM	CM-PLGA microspheres and IR820 coloaded methylcellulose hydrogel	[128]
	CM/PDA/SF/nHA	PDA—PTT CM—CMT	CM/SF/nHA	SF/nHA scaffold with CM-modified PDA nanoparticles	[129]
	AKT/SA/Pluronic F-127/Fe_3_S_4_	Fe_3_S_4_—MHT Fe—CDT	AKT/SA	AKT scaffold with Fe_3_S_4_ loaded surface coating.	[131]
	BG/FeSAC	FeSAC—PTT FeSAC—CDT	BG	BG scaffold incorporated with FeSAC	[134]
	MSN/Cu_2_S/oDEX	Cu_2_S—PTT Cu—CDT Cu—Copper death	Cu	Cu_2_S@BSA-loaded MSN/oDEX nanoparticles via Schiff base bonding.	[138]
	Cu-HHTP/PCL	Cu-HHTP—PTT Cu—CDT	Cu	PCL scaffold with Cu-HHTP loaded surface coating.	[137]
	Fe_3_O_4_/HA/SiO_2_/PLMC	Fe_3_O_4_—PTT Fe_3_O_4_—MHT Fe—CDT	HA	HA/SiO_2_/PLMC scaffold with Fe_3_O_4_ loaded surface coating.	[16]
	Ti/LDH/MgO-FeOx	FeOx—PTT FeOx—PDT	MgO	Ti with MgO/FeOx nanosheet loaded surface coating.	[20]
	IR780/HMnO_2_	IR780—PTT IR780—PDT Mn^4+^—Deplete glutathione	HMnO_2_	IR780-loaded hollow HMnO_2_ nanoparticles camouflaged with M1 macrophage membrane.	[141]
	MS**/**g-C_3_N_4_/MXenes/nHA	g-C_3_N_4_/MXenes—PTT g-C_3_N_4_/MXenes—PDT nHA—Mitochondrial dysfunction induction	nHA	MS scaffold incorporated with g-C_3_N_4_, MXenes and nHA.	[143]
	nHA/Au/CSMA/GelMA	Au—PTT nHA—Mitochondrial dysfunction induction	nHA	CSMA/GelMA hydrogel incorporated with nHA and Au nanorods.	[145]
	CR780-PEG/SA/PLLA/nHA	CR780-PEG—PTT nHA—Mitochondrial dysfunction induction	nHA	Bilayered hydrogel comprising an SA-based composite upper layer embedded with CR780-PEG/nHA and a PLLA-based lower layer loaded with nHA.	[144]
	ZIF-8/Ti_6_Al_4_V/DOX/IDO	ZIF-8—MTT DOX—CMT IDO—IMT	ZIF-8	Ti scaffold incorporated with IDO inhibitor-loaded ZIF-8@DOX.	[146]
	Nb_2_C MXene/MSN/R-SNO/BG	Nb_2_C MXene—PTT R-SNO—DNA damage induction	R-SNO/BG	BG scaffold incorporated with R-SNO-loaded MSNs silica-coated 2D Nb_2_C MXene.	[149]
	Zn/Li/Ti	Zn—CMT Li^+^—Blocking GSK-3 activation	Zn/Li	Ti scaffold incorporated with Zn and Li.	[150]
	CS/ZIF-8/DOX/PD-L1 siRNA/PCL	DOX—CMT PD-L1—siRNA IMT	ZIF-8	CS/PCL hydrogel incorporated with ZIF-8/DOX/PD-L1 siRNA/PCL	[151]

### Unitary anticancer optimization-based bifunctional strategic materials

#### Unitary hyperthermia optimization-based bifunctional strategic materials

Hyperthermia has emerged as a promising antitumor modality; however, in response to the important damage induced by hyperthermia, tumor cells may substantially up-regulate their metabolism, particularly glycolysis. This metabolic shift generates an abundance of energy that supports the expression of heat shock proteins (HSPs), triggering a cytoprotective response that shields tumor cells from the effects of elevated temperatures [90]. To address this, photothermal antitumor therapy can be employed in conjunction with HSP70 inhibitors. However, the clinical application of HSP inhibitors is hindered by their low solubility, marked cytotoxicity, and rapid clearance [91].

**Fig. 5.**
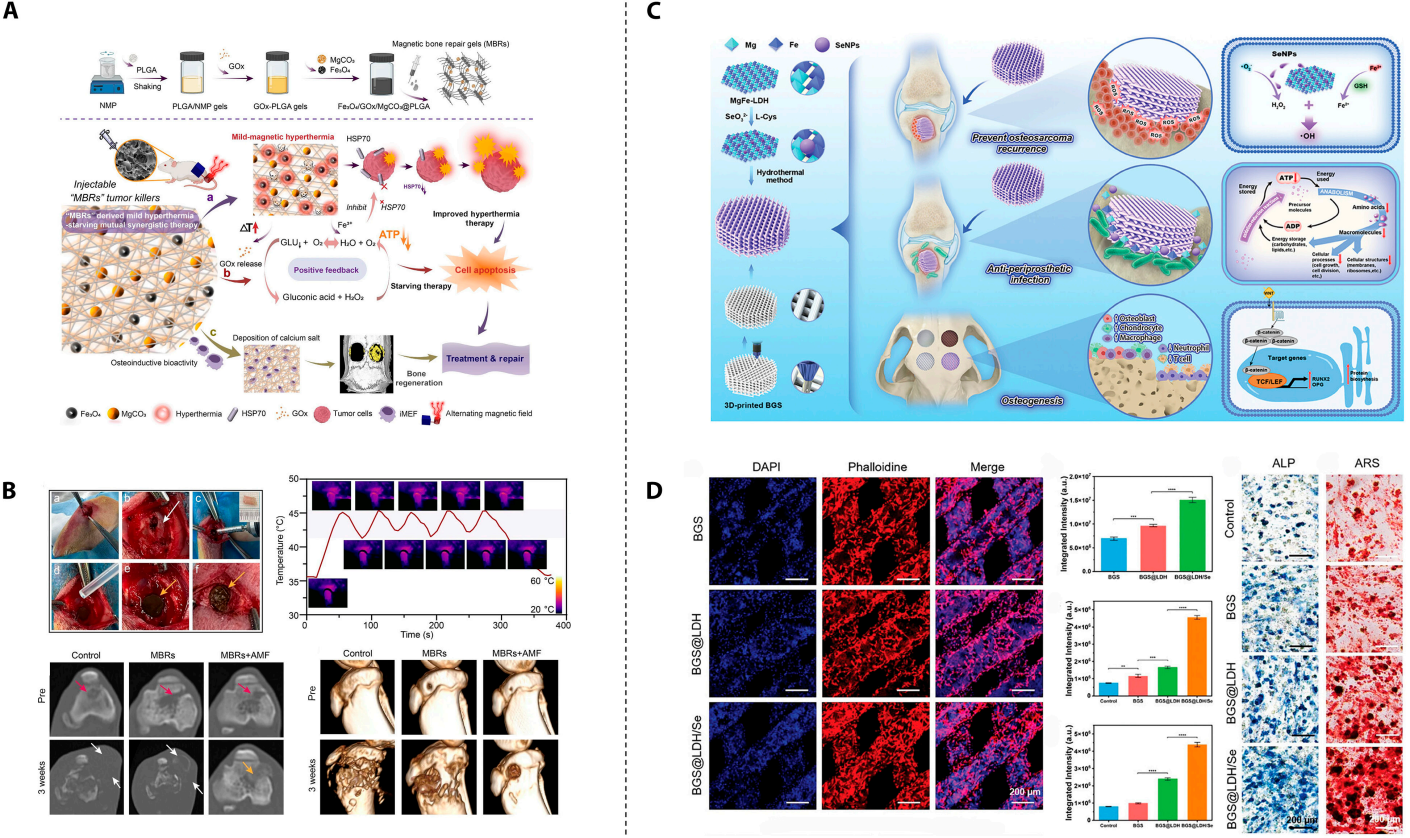
Unitary anticancer optimization-based bifunctional strategic materials. (A) Schematic illustration of the mechanism by which injectable multifunctional magnetic bone repair hydrogels (MBRs). (B) Evaluation of the MBRs’ antitumor ability for bone tumor in situ. Reproduced with permission [95]. Copyright 2023, Springer Nature. (C) A multifunctional SeNPs-incorporated MgFe-LDH nanosheets platform for a “one-stop-shop” strategy. (D) In vitro osteogenic efficacy evaluation of the composite material. Reproduced with permission [105]. Copyright 2024, John Wiley and Sons.

The primary proposed mechanism involves suppressing HSP expression through metabolic modulation to disrupt energy supply for HSP synthesis. The alleviation of oxygen deficiency may suppress PTT-induced HSP expression, based on the fact that glycolysis occurs primarily under hypoxic conditions. Xu et al. [92] developed a polycaprolactone (PCL)/nano-hydroxyapatite (nHA)/MgO_2_/PDA scaffold using 3D printing technology, which uniformly distributed MgO_2_ powder throughout the scaffold material. The MgO_2_ underwent a slow reaction with water, enabling the continuous release of oxygen. The provision of oxygen serves a dual purpose: it potentiates PDA-based PTT by inhibiting HSP expression and ameliorates the hypoxic tumor microenvironment to facilitate subsequent repair processes. Additionally, PCL, nHA, and magnesium ions provide sufficient mechanical support and induce osteogenesis. However, studies have found that tumors can also undergo glycolysis in an oxygen-rich environment, particularly in the case of solid tumors such as osteosarcoma [93,94]. Therefore, more effective methods are still needed to inhibit the expression of HSPs and thereby enhance the therapeutic efficacy of PTT. The fundamental mechanism underlying glucose oxidase (GOx)-mediated starvation therapy involves converting intratumoral glucose and oxygen into gluconic acid and hydrogen peroxide, aiming to reduce the tumor’s energy supply and inhibit the production of HSPs. However, this process is substantially hindered by the tumor’s hypoxic microenvironment. To address this challenge, Yu et al. [95] developed a novel multifunctional hydrogel by encapsulating magnetic Fe_3_O_4_ nanoparticles, MgCO_3_ particles, and GOx within an injectable PLGA hydrogel (Fig. 5A). In order to prevent potential damage to surrounding healthy tissues and to mitigate GOx inactivation due to high temperatures, the therapeutic temperature was maintained between 40 and 45 °C. The presence of Fe^3+^ ions, along with the controlled temperature, acted as a catalyst to facilitate the breakdown of H_2_O_2_ into H_2_O and O_2_. This catalytic reaction not only generated positive feedback by replenishing oxygen but also consumed substantial amounts of glucose. This method successfully creates a synergistic effect between mild hyperthermia and starvation therapy (Fig. 5B), while additional components such as magnesium ions further promote bone regeneration.

An alternative strategy involves direct structural disruption of HSP proteins. Single-atom nanozymes (SAzymes) are capable of generating large amounts of ROS, which react with the primary amines of HSPs, disrupting their structure and function [96]. Yan et al. [97] synthesized a single-atom copper nanozyme by selectively etching the atomic-level mixed A-layers (Al and Cu) from a quaternary MAX phase [Ti_3_(Al1-xCux) C_2_]. The SA-Cu-MXene was then incorporated into a poly-L-lactic acid (PLLA)/BG matrix to create a novel composite artificial bone scaffold. This scaffold exhibits photothermal conversion properties and can enhance lipid peroxidation and ROS production, ultimately leading to ferroptosis and the inactivation of HSP70. The composite bone scaffold promotes bone regeneration under physiological conditions through the sustained release of bioactive ions (Ca^2+^, P^5+^, Si^4+^, and Cu^2+^).

A third strategy employs epigenetic modulation to enhance tumor susceptibility to therapy. The lysine demethylase KDM3A can demethylate p53-K372me1, inhibiting the tumor-suppressive functions of p53, which, in turn, down-regulates the expression of phorbol-12-myristate-13-acetate-induced protein 1 (PMAIP1) and p53 up-regulated modulator of apoptosis (PUMA), leading to tumor resistance to PTT [98]. Consequently, the down-regulation of KDM3A increases the epigenetic sensitivity of tumors to PTT. 5-Carboxy-8-hydroxyquinoline (IOX1), an inhibitor of histone demethylase KDM3A, has been explored to achieve this effect. Liang et al. [99] modified ultrathin silicene nanosheets (NS) onto a 3D-printed BG scaffold and administered an intraperitoneal injection of IOX1 20 h before scaffold implantation surgery in mice, which substantially enhanced the efficacy of PTT. Calcium, silicon, and phosphates released during the degradation of scaffolds promote osteogenesis by stimulating the proliferation, differentiation, and mineralization of bone marrow mesenchymal stem cells.

#### Unitary CDT optimization-based bifunctional strategic materials

While CDT efficacy critically depends on ROS accumulation, 2 fundamental barriers compromise its potency: (a) antioxidant defense via glutathione (GSH)-mediated ROS scavenging [100], and (b) insufficient intratumoral H_2_O_2_ substrate [101]. Liu et al. [102] pioneered mixed-valence vanadium (IV/V)-doped mesoporous BG nanoparticles synthesized via microemulsion-assisted sol-gel, establishing self-circulating ROS amplification for tumor therapy. The vanadium (V)/vanadium (IV) redox cycle consumes GSH to generate cytotoxic ROS and regenerate pentavalent vanadium, disrupting tumor redox homeostasis while released silicon/calcium/phosphorus/vanadium ions synergistically promote bone marrow mesenchymal stem cell proliferation, osteogenic protein secretion, and mineralization.

Selenium nanoparticles (SeNPs) can activate superoxide dismutase, converting superoxide anion radicals (·O^2−^) into H_2_O_2_, thereby increasing H_2_O_2_ levels [103]. Layered double hydroxides (LDHs) are a class of 2-dimensional (2D) layered nanomaterials that have found extensive applications in biomedical fields such as drug delivery, cancer therapy, and tissue engineering due to their unique layered structure, large specific surface area, good biocompatibility, and biodegradability [104]. Bian et al. [105] developed a self-renewing H_2_O_2_ supply strategy by synthesizing SeNPs/MgFe-LDH nanosheets via coprecipitation and integrating them onto 3D-printed BG scaffolds (Fig. 5C). In this system, Fe^3+^ is reduced to Fe^2+^ by consuming GSH in the TME, triggering the Fenton reaction and generating ROS. Meanwhile, SeNPs supply additional precursors for the Fenton reaction, enhancing CDT efficiency and improving tumor cell killing. Additionally, the sustained release of Mg^2+^, Fe^3+^, and BG components promote increased bone mass (Fig. 5D).

### Multiple anticancer therapeutics-based bifunctional strategic materials

Beyond optimizing monotherapies, the synergistic integration of multiple antitumor treatment modalities represents another crucial strategy to enhance therapeutic efficacy and overcome drug resistance. This is not a mere superposition of treatments, but rather a synergistic effect where “1 + 1 > 2” [106].

#### PTT + CMT-based bifunctional strategic materials

The synergistic effect of combining PTT and CMT is multilevel, primarily manifested in these aspects: First, local hyperthermia enhances the fluidity of tumor cell membranes, promotes the endocytosis of chemotherapeutic drugs, and thereby increases intracellular drug concentrations [107]. Second, the high temperature generated by PTT can inhibit the DNA repair mechanism of cancer cells, making them more susceptible to the cytotoxic effects of chemotherapeutic drugs. Third, this combined treatment approach strengthens the body’s immune response, activating the immune system’s capacity to recognize and eliminate cancer cells [108]. In hydrogel systems, Li et al. [109] loaded BP nanosheets (photothermal agents) and DOX into injectable CS hydrogels, achieving enhanced tumor killing via PTT-CMT synergy and bone regeneration through BP-derived phosphate ions. Liu et al. [110] developed GelMA/oxidized dextran/MMT-Sr/Ppy hydrogels: their dual-network structure sustained DOX release, Ppy enabled high photothermal conversion, and MMT-Sr improved mechanics and osteogenesis. Chen et al. [111] coloaded PDA-encapsulated DOX and Sr ions in alginate/GelAGE hydrogels, utilizing CMT-PTT synergy for osteosarcoma elimination and Sr ions for bone formation. Yao et al. [112] developed an injectable SOH1(CP)1 hydrogel system—composed of sericin, oxidized chondroitin sulfate, Se/Mg-doped HAp nanorods, and PDA-coated CaO₂ nanospheres—to address incomplete osteosarcoma resection and bone defects. The hydrogel eradicates tumors through mild PTT and SeO_3_^2-^ CMT (achieving 100% inhibition at 18 days), while its degradation–matching repair cycle releases osteogenic ions (Ca^2+^, Mg^2+^, and PO₄^3−^) to fully repair bone defects within 12 weeks. This dual-functional system provides a new solution for osteosarcoma-related bone reconstruction.

Scaffold platforms integrate advanced fabrication with multifunctionality. Zhu et al. [113] 3D-printed polyetheretherketone (PEEK)/graphene scaffolds coated with CDDP-loaded HA, combining graphene-mediated PTT with chemo-bioactive antitumor effects. Rezk et al. [114] conjugated DOX with nHA via solution intercalation, electrospun DOX-loaded nHA/PCL nanofibers, and PDA-coated scaffold, yielding multifunctional photothermal/chemotherapeutic/osteogenic properties. Wang et al. [115] used low-temperature printing with a nano-emulsion (PLGA/β-TCP/BP/DOX/P24 peptide), where BP prolonged degradation for sustained PTT and P24 peptide synergized with degrading BP/β-TCP for bone repair. Bioinspired designs like Wang et al.’s [116] PDA/CDHA/DOX scaffolds achieved NIR-responsive PTT, controlled CMT, and bone-mimetic osteogenesis/angiogenesis. Rezk et al. [117] engineered GO-doxorubicin-whitlockite composites (WG@DOX) with pH/NIR-responsive release and photothermal properties. The platform synergized DOX CMT and GO hyperthermia to eradicate osteosarcoma cells, while GO/Mg^2+^ enhanced osteogenic activity via up-regulated protein expression. To address chemo-induced immunosuppression, Yan et al. [118] grafted low-toxic Pt (IV) onto PDA (PDA@Pt) and incorporated it into PLLA/BG scaffolds. This enabled on-demand Pt (II) release for low-toxic CMT, synergizing with PTT to eradicate tumors, activate cGAS-STING (converting “cold” to “hot” tumors), and promote osteogenesis.

Compared to traditional chemotherapeutic drugs, natural compounds with antitumor properties have gained important attention due to their reduced side effects. However, given their limited efficacy when used alone, they are frequently administered in combination with hyperthermia therapy. Chlorogenic acid (CA), a typical phenolic acid, exhibits not only antitumor activity but also osteogenic potential [119,120]. Yang et al. [121] grafted CA onto gold (Au) nanorods via gold-catechol bonds, which demonstrated significant osteosarcoma inhibition and bone regeneration activity under controlled hyperthermic conditions. Quercetin (Qr), a flavonoid compound, exhibits antitumor growth and bone regeneration-promoting activities [122,123]. Mesoporous silica nanoparticles (MSNs) possess excellent biocompatibility, high drug loading capacity, and controlled drug release properties [124]. Shi et al. [125] synthesized a multifunctional nanomaterial using MSN as a platform, incorporating Qr, collagen (Col), and PDA modifications. PDA’s photothermal effect synergizes with Qr’s antitumor activity, while Qr collaboratively promote bone regeneration. Curcumin (CM), a natural phenolic compound prevalent in rhizomes such as turmeric, mustard, curry, and saffron, has demonstrated anti-osteosarcoma activity and bone regeneration-promoting effects [126,127]. Tan et al. [128] encapsulated CM microspheres within an IR820-conjugated methylcellulose hydrogel, while Han et al. [129] developed a fibrous scaffold composed of CM, PDA nanoparticles, silk fibroin (SF), and nHA. Both systems exhibit chemo-photothermal synergistic anti-osteosarcoma effects and induce bone defect regeneration.

#### PTT or MHT + CDT-based bifunctional strategic materials

Hyperthermia combined with CDT represents another highly promising therapeutic paradigm. High temperature plays multiple complementary roles in enhancing CDT. First, elevated temperature substantially accelerates the kinetics of Fenton or Fenton-like reactions catalyzed by transition metal ions, thereby boosting the generation of highly cytotoxic hydroxyl radicals. Second, high temperature has been shown to sensitize intracellular organelles such as lysosomes and endoplasmic reticula, rendering them more vulnerable to ROS-induced damage [130]. Iron-based and copper-based systems have emerged as 2 major strategies to achieve synergistic antitumor effects through distinct mechanisms. Zhuang et al. [131] developed a novel approach by coating 3D-printed akermanite (AKT) bioceramic scaffolds with a layer of ferromagnetic Fe_3_S_4_ nanoparticles possessing a tunable microstructure. This Fe_3_S_4_ layer exhibits a magnetothermal effect, allowing for localized heating under an applied magnetic field. Simultaneously, the Fe^3+^ ions facilitate Fenton-like reactions, contributing to the cytotoxic effects. Importantly, the appropriate thermal energy generated by the magnetic hyperthermia also enhances the efficacy of the CDT, resulting in a synergistic elimination of residual tumor cells by the combined magnetic hyperthermia and CDT approach. Single-atom iron catalysts, composed of an amorphous carbon matrix anchoring individual iron atoms, exhibit multifaceted functionalities, including prominent light absorption in the NIR-I window and Fenton-like activity [132,133]. Wang et al. [134] incorporated highly active single-atom iron catalysts (FeSACs) into a BG scaffold, endowing it with unique capabilities. This design endows the scaffold with dual capabilities: ROS generation for CDT and NIR photothermal conversion for tumor ablation, while simultaneously enhancing antibacterial efficacy to create a favorable microenvironment for bone repair.

**Fig. 6.**
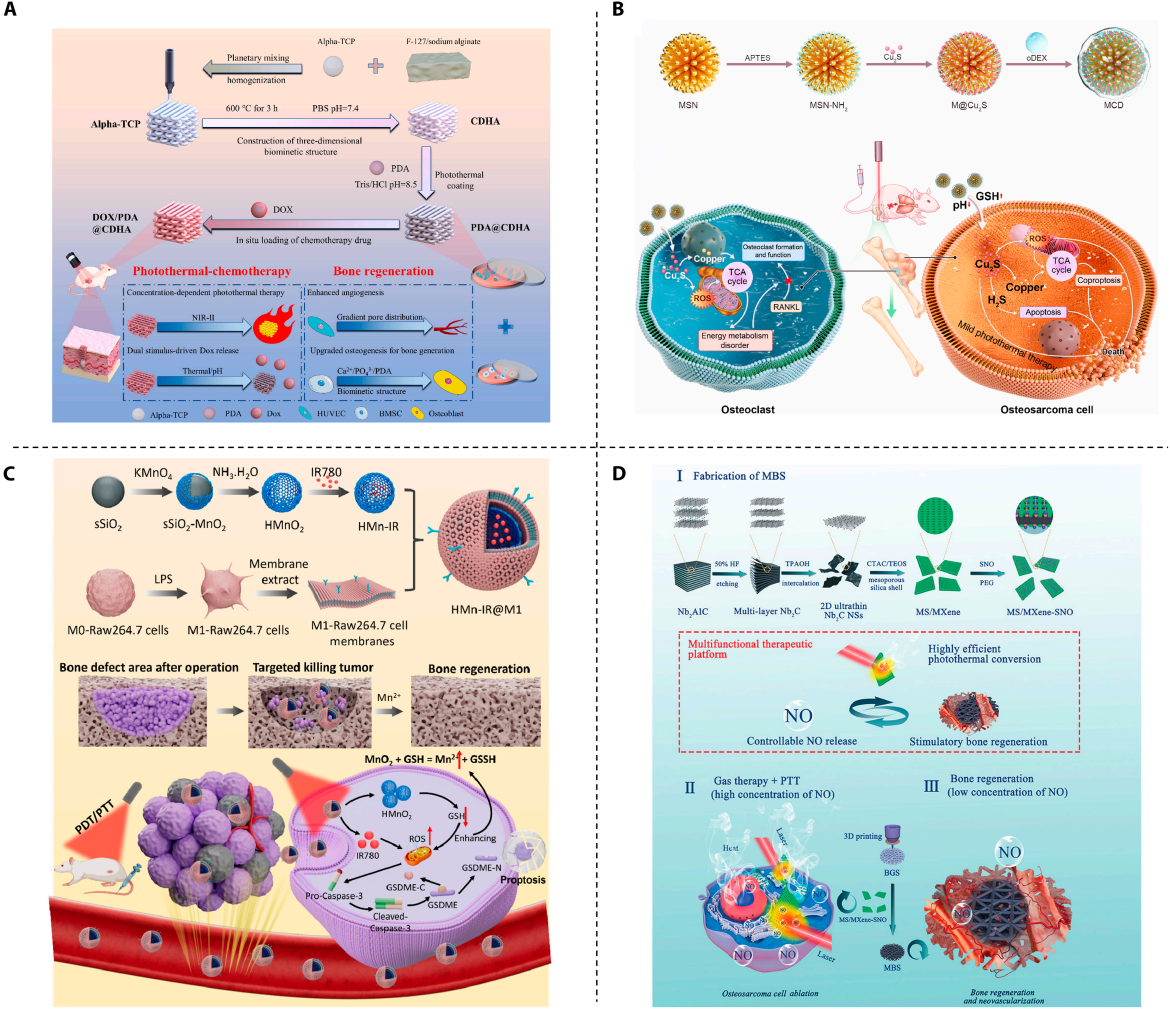
Multiple anticancer therapeutics-based bifunctional strategic materials. (A) Schematic illustration of PDA-coated 3D printed CDHA scaffolds with NIR-triggered drug release of DOX for synergistic PTT/CMT of osteosarcoma. Reproduced with permission [116]. Copyright 2024, Elsevier. (B) Schematic illustration of the MCD nanoparticles for therapy of osteosarcoma. Reproduced with permission [138]. Copyright 2024, Elsevier. (C) Synthesis concept of HMn-IR@M1 and applied in tumor targeted therapy and tissue regeneration of tumor bone defect. Reproduced with permission [141]. Copyright 2024, American Chemical Society. (D) Schematic of a multifunctional scaffold enabling controlled NO release, synergistic photothermal conversion, and osteogenic activation for combinatorial bone tumor therapy and regeneration. Reproduced with permission [149]. Copyright 2020, John Wiley and Sons.

Copper ions can directly disrupt the tricarboxylic acid (TCA) cycle, thus persistently impairing mitochondrial metabolism [135]. Furthermore, copper ions can generate ROS through Fenton-like reactions [136]. Huang et al. [137] fabricated a 3D-printed PCL scaffold engineered with a nanostructured 2D copper-based conjugated metal-organic framework (MOF) composed of copper ions (Cu^2+^) and the conjugated organic ligand 2,3,6,7,10,11-hexahydroxytriphenylene (HHTP) for synergistic PTT and CDT treatment of osteosarcoma. Ye et al. [138] electrostatically decorated dendritic MSN with ultrasmall Cu_2_S@BSA hybrid nanoparticles. Subsequently, a Schiff base conjugation with oxidized dextran (oDEX) conferred pH-responsiveness to the material. The Cu_2_S nanoparticles induce mitochondrial dysfunction, ultimately leading to copper-mediated toxicity in tumor cells while simultaneously generating ROS. The material also exhibits photothermal therapeutic properties under NIR-II laser irradiation. Importantly, this composite nanomaterial promotes BMSC osteogenic differentiation while inhibiting osteoclastogenesis by suppressing the expression of FDX1, LIAS, and DALT proteins in the TCA cycle (Fig. 6B). Four-dimensional (4D) printing is an advanced manufacturing technology that builds upon 3D printing by introducing the dimension of time. It enables printed objects to autonomously change their shape, properties, or functions over time in response to specific external stimuli [139]. Wang et al. [16] further advanced this field porous Fe_3_O_4_ scaffold via Pickering emulsion templating, enabling multimodal therapy (CDT/PTT/MHT) and bone regeneration. Its photothermal-responsive shape memory effect allows precise adaptation to complex bone defects for minimally invasive implantation, while interconnected pores facilitate osteogenic differentiation.

#### PTT + PDT-based bifunctional strategic materials

Similar to the hyperthermia-CDT paradigm, the combination of PDT and PTT importantly enhances tumoricidal efficacy by inducing severe organelle dysfunction, mitochondrial collapse, and activation of both apoptotic and immunogenic cell death pathways [140]. Bimetallic oxide systems, for instance, effectively integrate multimodal therapy with bone repair: Zhang et al. [20] constructed bilayer MgO/FeOx nanosheets on titanium implants, where the bimetallic structure endows dual capabilities of efficient photothermal conversion (for hyperthermia) and ROS generation (for PDT) to synergistically kill tumor cells, while releasing OH^-^ to neutralize acidic microenvironments and Mg^2+^ ions to enhance antitumor/antibacterial effects and promote angiogenesis and bone regeneration. Nanocarrier-based platforms further optimize targeted delivery and therapeutic synergy: Ma et al. [141] encapsulated IR780 in hollow manganese dioxide (HMnO_2_) nanocarriers modified with M1 macrophage-derived cell membranes for tumor site-specific accumulation (Fig. 6C), where IR780 mediates both PTT and PDT, Mn^4+^ depletes intracellular GSH to amplify ROS production and induce ferroptosis, and degraded Mn^2+^ by-products contribute to osteogenic regeneration. MXene-based composites, leveraging intrinsic photothermal activity and high electrical conductivity to facilitate photogenerated charge separation [142], represent another promising category: Zhang et al. [143] utilized melamine sponge (MS) as a framework to incorporate g-C_3_N_4_/MXenes composites with nHA, constructing a dual-functional scaffold where g-C_3_N_4_/MXenes drive synergistic PTT-PDT, while nHA enhances antitumor efficacy via mitochondrial dysfunction and promotes osteogenesis, thus realizing dual therapeutic and regenerative goals.

#### Other multiple anticancer therapeutics-based bifunctional strategic materials

Beyond the more common combinations like PTT + CMT or PTT + CDT, the strategy of “enhanced antitumor effect” is further exemplified by innovative material designs that integrate distinct therapeutic modalities with unique synergistic mechanisms. These approaches often leverage the intrinsic properties of biomaterials to create multifaceted antitumor microenvironments or engage novel cell death pathways, thereby achieving a more potent and comprehensive eradication of residual tumor cells. Gong et al. [144] developed a biomimetic bilayer scaffold composed of a top layer of CR780-polyethylene glycol (PEG) conjugated SA composite hydrogel and a bottom layer of PLLA and nHA. The layers were interconnected via hydrogen bonding and interfacial reactions. This biomimetic bilayer scaffold provided a porous structure and sufficient mechanical strength for osteoblasts and chondrocytes, while the photothermal effect and nHA-induced mitochondrial dysfunction synergistically induced apoptosis of residual tumor cells. Liao et al. [145] incorporated gold nanorods and nHA into a mixed hydrogel composed of GelMA and chondroitin sulfate methacryloyl (CSMA). The composite hydrogel, under dual stimuli of photothermal irradiation and nHA, eliminated residual tumor cells after surgery. Simultaneously, the hydrogel filled the bone defect and, in conjunction with nHA, effectively promoted bone formation. Ma et al. [146] developed a multifunctional scaffold for combined MTT, CMT, IMT, and bone integration. ZIF-8, an MOF with microwave chemosensitivity, was used as a delivery vehicle for DOX and indoleamine 2,3-dioxygenase (IDO) inhibitors, and integrated into a 3D-printed porous titanium scaffold. This synergistic approach not only effectively eliminates tumor cells but also accelerates bone regeneration through ZIF-8 degradation products. The scaffold provides sufficient mechanical support, and its porous structure creates space for bone growth. High concentrations of nitric oxide (NO) exhibit direct anticancer effects through multiple mechanisms, primarily involving DNA damage and the inhibition of DNA repair enzymes [147,148]. Yang et al. [149] coated 2D Nb_2_C MXene with MSN loaded with S-nitrosothiols (R-SNO) and then integrated this composite into a glass scaffold (Fig. 6D). The MXene component endowed the scaffold with PTT capabilities while simultaneously releasing high concentrations of NO, thereby synergistically killing tumor cells. On the other hand, at lower concentrations, NO can promote angiogenesis and, in conjunction with BG, facilitate bone regeneration. Lu et al. [150] developed 3D-printed Zn-0.8Li porous scaffolds that synergistically combat residual osteosarcoma while promoting bone regeneration post-resection. Through controlled corelease of Zn^2+^ (inhibiting tumor proliferation/migration via proteasome ubiquitination, ER stress, and DNase silencing) and Li^+^ (blocking GSK-3 activation), the scaffolds eliminate tumor cells while enhancing osteoblast differentiation. Zhou et al. [151] engineered a catechol-modified CS hydrogel (Gel@RDZ) encapsulating ZIF-8 nanoparticles coloaded with DOX and PD-L1 siRNA for postoperative maxillofacial osteosarcoma management. The hydrogel enables sustained local release of therapeutic agents to eradicate residual tumor cells via DOX CMT and PD-L1 blockade-enhanced CD8^+^ T cell immunity, while simultaneously promoting bone regeneration through scaffold-functionalized osteogenesis in critical-sized defects.

To address the clinical challenges of high malignancy and recurrence rates in osteosarcoma, this dual-function strategy prioritizes the enhancement of tumor-killing efficacy. By improving the thermal sensitivity of materials, boosting ROS generation efficiency, or synergistic multiple modalities such as CMT and PTT, it substantially strengthens the ability to eliminate residual tumor cells. However, while optimizing tumor eradication, careful consideration must be given to whether excessively potent antitumor effects may cause damage to surrounding tissues, thereby adversely impacting bone healing.

## Bifunctional Strategies Based on Temporal Regulation

The prevailing research currently focuses on the direct integration of multifunctional materials with antitumor and osteogenic properties, often neglecting the temporal dimension of therapeutic needs. Premature promotion of bone tissue regeneration increases the risk of tumor recurrence and metastasis. Activation of bone formation-related pathways such as Wnt, Hedgehog, and Tgf-β is intimately linked to the progression of osteosarcoma [152–154]. In contrast, prolonged antitumor treatments can cause excessive damage to normal cells, adversely affecting bone healing in later stages of therapy. Therefore, a sequenced therapeutic strategy based on temporal regulation antitumor promotion and osteogenesis is garnering increasing attention (Table 3).

**Table 3.** Bifunctional strategic materials based on temporal regulation

Strategy	Biological materials	Antitumor	Osteogenesis	Specific details	Ref.
Prolonged osteogenesis time	BP-HyA/αPD-L1/vismodegib/Mg	αPD-L1—IMT Vismodegib—IMT	Mg	HA-BP-Mg hydrogel incorporated with vismodegib and αPD-L1. α PD-L1/vismodegib release ends in 2 weeks. Mg ion release >3 weeks.	[156]
	nHA/Cu/PLA/Sa/GelMA	CU—CDT	nHA/Cu	Acidic TME rapidly releases CCP, depleting GSH and generating lethal ROS via Cu^+^-mediated Fenton reaction. Post-eradication, normalized pH enables sustained Cu^2+^/nHA release, stimulating osteogenic differentiation.	[157]
	MgO_2_/PLGA	MgO_2_—CDT	Mg	PLGA hydrogel incorporated with 20 wt% MgO_2_. ROS release ends in 3 weeks. Mg ion release in 12 weeks.	[158]
	Gel/PDA/MgO_2_	PDA—PTT MgO_2_—CDT	Mg	GelDA hydrogel incorporated with 10 mg/ml MgO_2_. H_2_O_2_ release ends in 3 days. PTT for 7 days. Mg ion release >7 weeks.	[159]
Core–shell	HyAMel/F127DA/GelMA/Melatonin	Melatonin—CMT	Melatonin	HyAMel/F127DA core hydrogel incorporated with high-concentration melatonin, GelMA shell hydrogel with low-concentration melatonin. The shell releases high-concentration melatonin first, then the core releases low-concentration after shell degradation.	[165]
	CHW/PEGDA/GT/GG/DOX/magnesium-iron LDH	Magnesium-iron LDH—PTT DOX—CMT	CHW/PEGDA/magnesium-iron LDH	GT/GG shell hydrogel incorporated with LDOX, CHW/PEGDA core hydrogel with LDH. The shell hydrogel eliminates tumors via PTT/DOX synergy; after degradation, the core hydrogel promotes osteogenesis through mild photothermal, liquid crystal properties, and metal ions.	[167]
External switch controls	GA/cell-free bone matrix	GA—PTT	GA/cell-free bone matrix	GA-chemically modified cell-free bone matrix. High-power NIR induces thermal ablation for tumor eradication; reduced power enables mild photothermal activation to enhance osteogenesis	[173]
	GeSe/PLA/TCP	GeSe—PTT	GeSe/TCP	TCP scaffold with PLA/GeSe nanofiber films loaded surface coating. Sequential NIR-photothermal tumor ablation followed by ultrasound - electro stimulated osteogenesis.	[178]
	Bi/SrTiO_3_/Gelatin/OCS	Bi/SrTiO_3_—PDT Bi/SrTiO_3_—Increased PDT efficiency	Bi/SrTiO_3_	Gelatin/OCS hydrogel incorporated with Bi/SrTiO_3_ nanoheterostructure. US-NIR-boosted PDT rapidly ablates osteosarcoma via ROS storm, followed by NIR-off ultrasound-activated piezoelectric osteogenesis.	[181]
	BP/GSNO/BG	BP—SDT GSNO—DNA damage induction	BP/BG	US-activated and EGCG-induced pyroptosis and ROS. US off, phosphate and NO boost angiogenesis and bone repair.	[21]
	TiO-Ir/HA	TiO-Ir—SDT TiO-Ir—Mitochondrial damage		US-activated ROS in tumors, depletes GSH. Post-eradication, it converts H_2_O_2_→O_2_ in bone defects, suppressing oxidative stress and promoting osteogenesis.	[182]
	EGCG/SrTiO_3_/BG	SrTiO_3_—SDT SrTiO_3_—PANoptosis ECGG—DNA methylation reversal.	Sr/BG	US-activated and EGCG-induced pyroptosis and ROS. Sr in BG scaffolds post-US enhances BMSC osteogenesis for bone repair.	[183]
	BaTiO_3_/Ti	BaTiO_3_—Disrupted tubulin	BaTiO_3_	Ti scaffold with BaTiO_3_ ferroelectric nanorod array loaded surface coating. High-intensity US electrotherapy ablates tumors, followed by endogenous field-guided osteogenesis upon US cessation.	[185]

### Chronotherapeutic bone regeneration outlasts oncotherapeutic efficacy-based bifunctional strategic materials

**Fig. 7.**
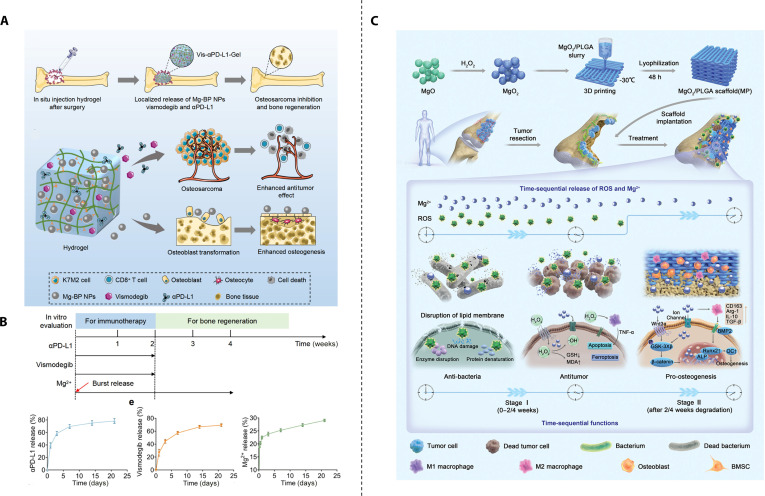
Chronotherapeutic bone regeneration outlasts oncotherapeutic efficacy-based bifunctional strategic materials. (A) Schematic illustration of bioactive nanocomposite hydrogel for enhanced bone tumor immunotherapy and bone regeneration. (B) The release profile of αPD-L1, vismodegib, and Mg^2+^ in vitro. Reproduced with permission [156]. Copyright 2024, Elsevier. (C) Schematic representation of the pathophysiology-based design concept of innovative MgO_2_/PLGA scaffolds. Reproduced with permission [158]. Copyright 2024, John Wiley and Sons.

A straightforward approach to achieve temporal control is by extending the release duration of osteoinductive substances. Indeed, numerous studies employ this strategy, such as bone bio-scaffolds loaded with antitumor drugs, where scaffold degradation occurs at a substantially slower rate than drug release. However, most of these studies fail to clearly define and distinguish between the antitumor phase and the osteoinductive phase, and therefore are not included in this section. Here, we select a few representative examples for presentation. Clinically, the efficacy of PD-1 monoclonal antibody treatment is suboptimal. One possible factor is that cancer-associated mesenchymal stem cells (CA-MSCs) drive CD8^+^ T cell tumor immune exclusion and reduce the response to anti-PD-L1 therapy. However, research has demonstrated that hedgehog pathway antagonists can reverse the effects of CA-MSCs, restore responsiveness to immune checkpoint blockade (ICB) treatment, and modulate antitumor immunity [155]. Chu et al. [156] engineered a bisphosphonate-modified hyaluronic acid (BP-HyA) hydrogel to sequentially deliver αPD-L1 and the hedgehog pathway antagonist vismodegib in the early IMT phase, followed by sustained release of Mg^2+^ ions during the subsequent bone formation phase. This strategy leverages differential molecular interactions and diffusion kinetics within the hydrogel network to achieve temporally controlled therapeutic actions (Fig. 7A). The hydrogel, injected into the bone defect after primary tumor resection, can sustainably prolong the release of αPD-L1 and vismodegib for up to 2 weeks, enhancing the activity of activated CD8^+^ T cells that have been suppressed by invading tumor cells. Simultaneously, the release duration of magnesium ions is longer than that of αPD-L1 and vismodegib, lasting for more than 3 weeks (Fig. 7B), and the initially released Mg^2+^ contributes to the enhancement of CD8^+^ T cell activation.

Xie et al. [157] engineered a time-sequential 3D-printed poly lactic acid (PLA)/nHA scaffold integrated with pH-responsive hydrogel to deliver tumor-targeting Cu–Cys–PEG nanoparticles (CCP). During the initial phase, the acidic tumor microenvironment triggers hydrogel disintegration and rapid release of copper nanoparticles, inducing CDT. In the subsequent phase, following tumor elimination and pH normalization, the system switches to sustained copper ion release that synergizes with nHA to promote osteogenic differentiation, thereby enabling continuous bone regeneration. Li et al. [158] utilized low-temperature 3D printing to fabricate a layered porous nanocomposite scaffold composed of magnesium peroxide (MgO_2_) and PLGA, enabling the controlled sequential release of ROS and magnesium ions (Mg^2+^) (Fig. 7C). The degradation of MgO_2_ occurs in 2 stages: first, MgO_2_ decomposes into magnesium hydroxide [Mg (OH)_2_] and hydrogen peroxide (H_2_O_2_) according to the reaction MgO_2_ + 2H_2_O → Mg(OH)_2_↓ + H_2_O_2_↑; next, Mg (OH)_2_ undergoes hydrolysis to release Mg^2+^ and hydroxide ions (OH^−^) following the reaction Mg(OH)_2_ → Mg^2+^ + 2OH^−^. MgO_2_ provides sufficient H_2_O_2_ for CDT while simultaneously releasing osteogenic Mg^2+^. The second stage of Mg^2+^ release is substantially slower than the first. Additionally, there is an initial burst release of Mg^2+^ due to the rapid degradation of Mg(OH)_2_ near the scaffold’s surface, while the Mg(OH)_2_ encapsulated deeper within the scaffold is gradually released as the PLGA degrades over time. This system achieves temporal control: during the first 3 weeks, H_2_O_2_ is released to initiate CDT in the antitumor phase, while from weeks 3 to 12, prolonged release of Mg^2+^ activates the Wnt3a/GSK-3β/β-catenin signaling pathway and creates an osteoinductive immune microenvironment through M2 macrophage polarization, thereby enhancing bone regeneration. The team also developed another spatiotemporally controlled hydrogel based on a similar principle. This innovative hydrogel leverages the reactivity of MgO₂ (which generates H_2_O_2_): in the presence of HRP, H_2_O_2_ triggers the oxidative polymerization of dopamine moieties on GelDA chains, thereby enabling hydrogel crosslinking and formation [159]. In addition to its PTT effects, the hydrogel releases H_2_O_2_ to trigger CDT, achieving synergistic tumor killing. Moreover, the sustained release of Mg^2+^ endows the hydrogel with time-controlled properties, further enhancing its therapeutic functionality.

### Core–shell sequential therapy-based bifunctional strategic materials

**Fig. 8.**
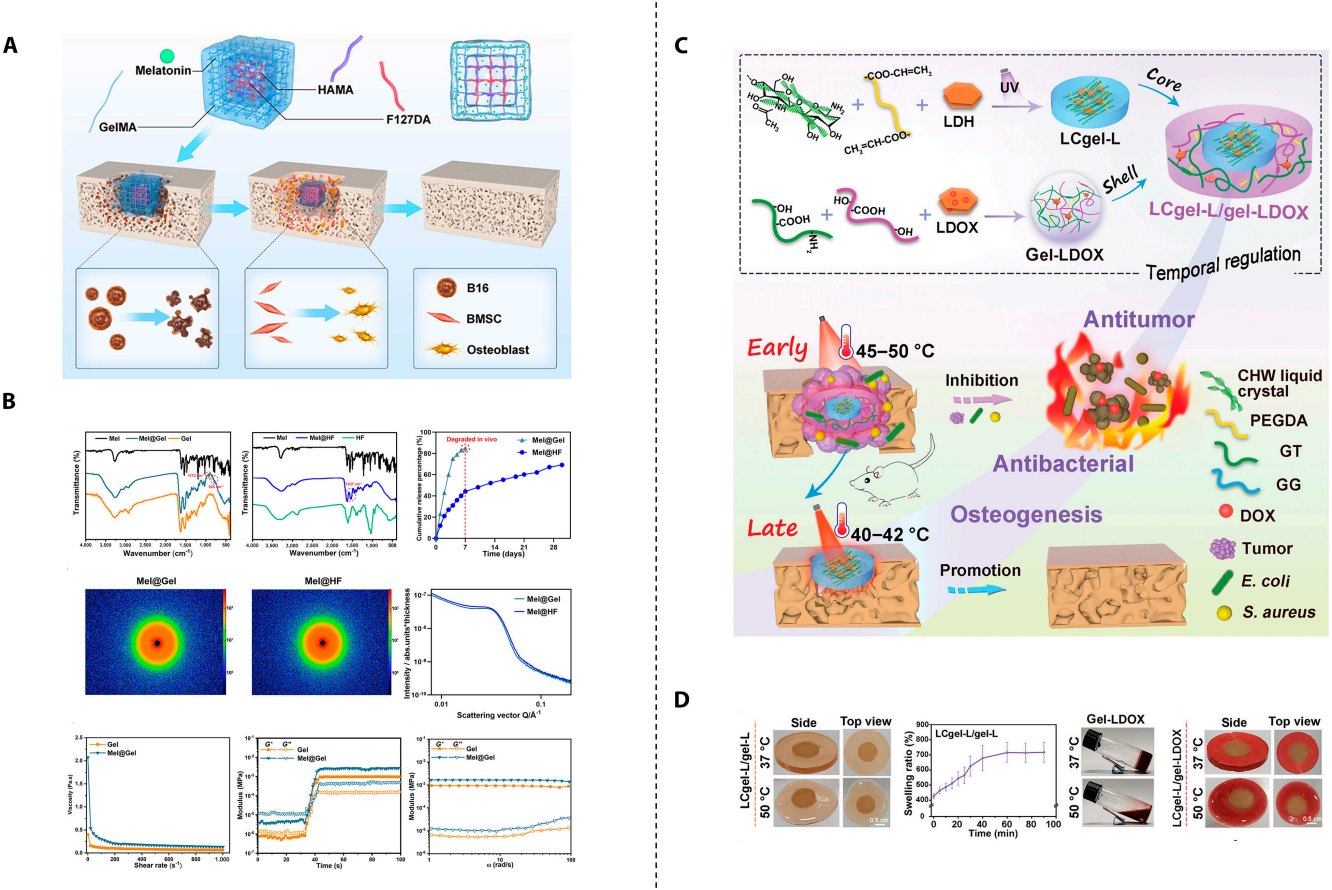
Core–shell sequential therapy-based bifunctional strategic materials. (A) Schematic illustration of Gel/HF for enhanced anti-osteosarcoma and bone regeneration. (B) Detection of melatonin loading and release as well as rheological properties of Mel@Gel/Mel@HF. Reproduced with permission [165]. Copyright 2023, American Chemical Society. (C) Schematic illustration of the construction of core–shell hydrogel of LCgel-L/gel-LDOX for achieving early antitumor and antibacterial and late osteogenic effects. (D) Hydrogel physicochemical and morphological properties under varying temperatures. Reproduced with permission [167].l Copyright 2024, Elsevier.

Another more effective approach to achieve temporal regulation is the core–shell structure. The core–shell structure provides sequential release: agents in the faster-degrading shell release first, followed by those in the slower-degrading core [160–162]. Melatonin is an endogenous neurohormone that has garnered attention for its dual role in inhibiting osteosarcoma and promoting osteogenesis [163]. However, while melatonin exhibits antitumor effects at high concentrations, it also inhibits bone regeneration at these levels. Only at low concentrations does melatonin promote bone regeneration [164]. This raises the question: how can melatonin’s concentration be modulated in osteosarcoma treatment to achieve temporal control for sequential antitumor and osteogenic therapy? Huang et al. [165] designed a core–shell hydrogel bone scaffold to address this issue. The core is composed of a composite of hyaluronic acid methacrylate (HyAMel) hydrogel and F127DA hydrogel, loaded with low concentrations of melatonin. After ultraviolet (UV) curing, the core forms a cubic structure. The shell, made of GelMA and containing high concentrations of melatonin, is slowly injected around the core. Following UV curing, it forms a shell encapsulating the core (Fig. 8A). The core–shell structure precisely controls melatonin release: high concentrations of melatonin are rapidly released in the early postoperative period (within 7 days) to eliminate residual tumor cells, while low concentrations are gradually released over the mid-to-late phase (up to 30 days) to promote osteogenesis (Fig. 8B). The viscoelastic and liquid crystal (LC) properties of bone-like materials play a critical role in osteogenesis. Liu et al. [166] developed a chitin whisker/polyethylene glycol diacrylate (CHW/PEGDA) LC hydrogel mimicking these characteristics and demonstrated its osteogenic potential in vivo. Building on this, Li et al. [167] designed a system where a UV-cured magnesium-iron LDH combined with an LC hydrogel formed the core, while a thermosensitive hydrogel shell—comprising gelatin (GT), gellan gum (GG), and DOX-loaded magnesium-iron LDH—was uniformly applied around the core (Fig. 8C). Magnesium-iron LDH is known for its excellent photothermal conversion efficiency in the NIR-II window [168], enabling it to generate heat for tumor ablation. Concurrently, the thermosensitive shell disintegrates under the heat, releasing DOX to synergistically eliminate residual tumor cells and bacteria through a combination of PTT and CMT (Fig. 8D). Recent studies have indicated that mild photothermal effects can enhance bone regeneration [169,170]. The core hydrogel, regulated by LDH to maintain a temperature of 40 to 42 °C, works in conjunction with the LC hydrogel and magnesium-iron ions to promote osteogenesis in the later stages of treatment.

### External control-based bifunctional strategic materials

**Fig. 9.**
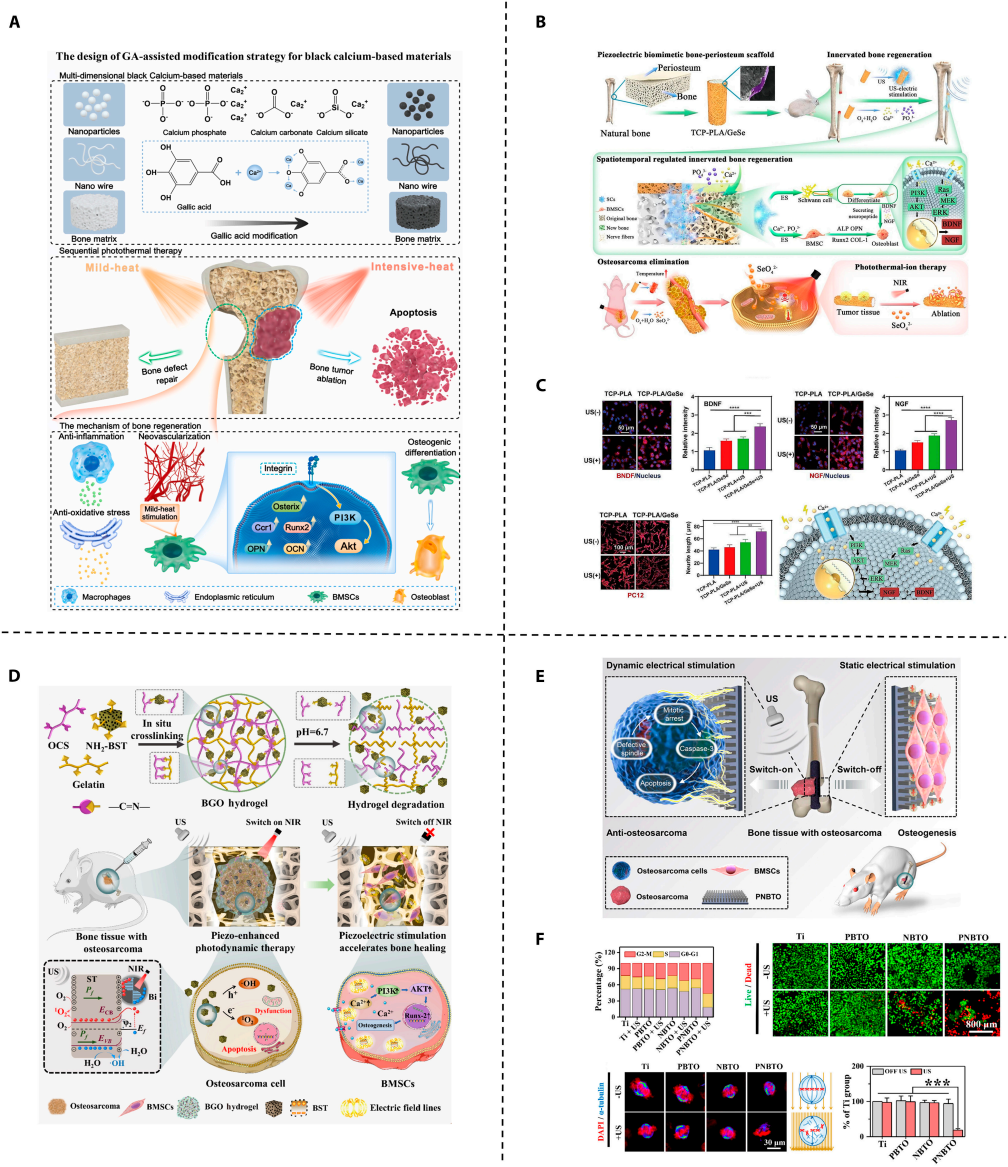
External control-based bifunctional strategic materials. (A) Schematic diagram of polyphenol-assisted modification strategy for bifunctional calcium-based materials preparation and of sequential PTT and osteogenesis. Reproduced with permission [173]. Copyright 2024, Elsevier. (B) Schematic illustration of the piezoelectric biomimetic bone-periosteum scaffold (TCP-PLA/GeSe) for spatiotemporal innervated bone regeneration and tumor ablation. (C) The neurogenic activity of SCs on TCP-PLA/GeSe scaffolds under US excitation. Reproduced with permission [178]. Copyright 2023, Springer Nature. (D) Piezoelectric field-driven fast charge separation coupling with surface plasmon resonance enhanced near-infrared photodynamic therapy for anti-osteosarcoma and osteogenesis. Reproduced with permission [181]. Copyright 2024, Elsevier. (E) One-dimensional ferroelectric nanoarrays with wireless switchable static and dynamic electrical stimulation for selective regulating osteogenesis and anti-osteosarcoma. (F) Dynamic electrical stimulation blocks the cell cycle and induces cell death in vitro. Reproduced with permission [185]. Copyright 2022, American Chemical Society.

An additional sophisticated approach to temporal regulation leverages externally controllable stimuli as a “switch”, allowing for precise, on-demand control of the timing for both tumor suppression and bone regeneration. By precise temperature regulation, it is possible to sequentially achieve tumor ablation followed by subsequent bone regeneration. Cell-free bone matrix is extensively employed for bone defects owing to its superior biocompatibility and osteogenic potential. However, the direct incorporation of photothermal agents imposes stringent requirements on the composition and structural integrity of biomaterials. Decellularized bone matrix is incompatible with this therapeutic strategy, thereby limiting its applicability in osteosarcoma treatment. Gallagic acid (GA), abundant in plants such as *Rheum palmatum*, *Eucalyptus robusta*, and *Cornus officinalis*, exhibits anti-inflammatory, antioxidant, and antitumor activities [171,172]. Its phenolic structure, featuring 3 hydroxyl groups, enables coordination with metal ions. Hou et al. [173] devised a universal modification strategy utilizing GA-assisted coordination chemistry to fabricate cell-free bone matrix with photothermal capabilities (Fig. 9A). GA modification confers pronounced photothermal properties, and NIR power modulation enables controlled temporal regulation—early high-temperature tumor ablation and late mild hyperthermia for bone regeneration—overcoming the incompatibility of decellularized bone matrix with traditional photothermal agents.

Endogenous bioelectricity is pervasive throughout the body’s physiological activities, and an increasing body of research indicates that modulating electrical signals through biomaterials can effectively stimulate the regeneration of bone, nerve, and other tissues [174,175]. Among these, piezoelectric materials—which convert mechanical stress into electrical energy to stimulate cells or influence their surrounding electric fields—are gaining important attention [176]. Ultrasound, with its excellent tissue penetration and noninvasive properties, is particularly well-suited as an external trigger for activating piezoelectric materials [177]. Xu et al. [178] integrated GeSe nanosheets, known for their remarkable piezoelectric properties and photothermal effects, into PLA to enhance both piezoelectric performance and photothermal functionality in a biomimetic periosteum (Fig. 9B). Using electrospinning, the nanofiber membrane was then coated onto a porous TCP scaffold. In the antitumor phase, NIR light stimulation triggered a photothermal response and the release of Ge ions, effectively destroying tumor cells. In the bone repair phase, without NIR stimulation, ultrasound irradiation generated electrical stimulation, which enhanced early neurogenic differentiation of Schwann cells and promoted osteogenic differentiation (Fig. 9C). This system provides precise temporal control, facilitating both tumor eradication and bone regeneration.

Piezoelectric materials also enhance PDT efficiency under ultrasound by accelerating photoinduced charge separation [179,180]. Xiao et al. [181] developed a piezoelectric–photonic nanostructure using ultrasmall bismuth/strontium titanate nanotubes (Bi/SrTiO_3_). These nanotubes exhibit localized surface plasmon resonance effects and a narrow bandgap, allowing them to respond to a broader range of wavelengths while possessing piezoelectric properties. These nanoparticles were integrated into a gelatin/sulfated chondroitin sulfate (OCS) injectable hydrogel. In the antitumor phase, when ultrasound and NIR light were simultaneously activated, the efficiency of PDT was importantly enhanced, leading to rapid production of ROS and effectively eradicating osteosarcoma cells. Furthermore, in the bone repair phase, the hydrogel exhibited excellent filling in the bone defect area. With the NIR light turned off, the hydrogel became polarized under ultrasound irradiation and transmitted electrical stimulation to cells, thereby exerting therapeutic effects on bone repair (Fig. 9D).

Piezoelectric materials can also be utilized for SDT to treat osteosarcoma. Fang et al. [21] engineered a 4D-bioactive BP-GSNO-BG scaffold, integrating black phosphorus nanosheets and NO donor GSNO for ultrasound-responsive osteosarcoma therapy. Ultrasound simultaneously triggers piezoelectric ROS generation from BP and bursts NO release from GSNO for tumor eradication, while later-stage degradation delivers phosphate ions and sustained low-dose NO to synergistically accelerate angiogenesis and bone regeneration. This dynamically transforming platform embodies biomaterial principles—transitioning from initial antitumor activity to long-term osteogenic function—enabling stepwise treatment of tumor resection defects. Rong et al. [182] developed intelligently sequential HS-ICTO scaffolds via 3D printing, integrating Ir-cluster-decorated TiO₂ (ICTO) biocatalysts on HA scaffolds for post-resection osteosarcoma therapy. Initially, ICTO generates massive ROS through sono-activation under tumor microenvironment, depletes GSH to disrupt redox homeostasis, and alleviates hypoxia via catalase-like O₂ production for enhanced tumor apoptosis. Following tumor elimination, as the acidic microenvironment shifts, the scaffold switches its function to catalyze H_2_O_2_ → O₂—suppressing oxidative stress while promoting stem-cell osteogenesis and inhibiting osteoclastogenesis—thereby enabling a seamless transition from tumor eradication to bone regeneration. Wang et al. [183] developed 3D-printed composite scaffolds featuring EGCG-loaded mesoporous SrTiO_3_ nanoparticles for logic-gated osteosarcoma therapy. The platform uses ultrasound-triggered piezoelectric ROS (“outer signal”) and tumor-acidity-activated EGCG-mediated DNA demethylation (“inner signal”) to induce PANoptosis, eradicating bacteria while activating antitumor immunity via dendritic cell maturation. Simultaneously, Sr^2+^/BG components promote bone regeneration through enhanced BMSC osteogenic differentiation.

Emerging studies reveal that intense electric fields can selectively exert direct cytotoxic effects on tumor cells [175,184]. Xiao et al. [185] engineered a ferroelectric BaTiO_3_ nanorod array coating for titanium implants. When activated with ultrasound, this coating produces high-intensity dynamic electric fields, fluctuating between −237 and 139 mV (Fig. 9E). These fields interfere with microtubule orientation, compromise spindle assembly, and halt mitosis at the G2/M phase, all of which contribute to tumor cell apoptosis (Fig. 9F). In the absence of ultrasound, a static electric field emerges from the interaction between the positively charged PNBTiO surface and the negatively charged bone defect. This electrostatic environment supports bone healing by fostering a conducive microenvironment for osseointegration. The application of electrical stimulation in the treatment of osteosarcoma remains an underexplored area, and there is a pressing need for robust, high-quality experimental evidence to substantiate its potential efficacy and safety.

This strategy achieves precise orchestration of the treatment sequence by modulating the release kinetics of osteogenic components, employing core–shell structures for temporally segregated functionality, or utilizing external remote-control switches to transition between “tumor-killing” and “bone-regeneration” phases. This temporal-control strategy ensures closer alignment with the pathophysiological process following surgery. Nevertheless, a central challenge remains: determining the optimal timing to deactivate the antitumor function and initiate the pro-regenerative phase. Future designs should focus on theranostic platforms integrating real-time diagnosis with therapeutic intervention for improved tumor surveillance.

## The Clinical Translation for Bifunctional Strategies

Despite the promising potential of bifunctional strategies in simultaneously addressing tumor eradication and bone regeneration, getting them from the laboratory to the clinic is proving to be a difficult journey. Several major hurdles still stand in the way. First, there are concerns about the long-term safety of these new materials; we need to be absolutely sure that they are not toxic to the body’s healthy tissues over time [186]. Besides long-term safety, another critical practical issue is closely linked to osteosarcoma surgery itself. Osteosarcoma surgery usually involves extensive resection and long operation time, which inherently raises the risk of postoperative infection. Similar to other orthopedic implants, implanting bifunctional materials may further increase this infection risk—this could not only affect treatment outcomes but also delay patient recovery [187]. Overcoming these practical obstacles is the essential next step before these innovative strategies can become a routine and reliable option for patients.

### Material toxicity assessment

Material toxicity is a major concern in biomedical applications, as toxic reactions can disrupt the body’s normal physiological balance, trigger inflammatory responses, and may even lead to long-term organ problems [28,186,188]. For instance, as frequently mentioned earlier, multiple metal ions (e.g., Mg, Zn, and Cu) are used for CDT or osteogenesis/angiogenesis. However, excessively rapid release of these ions can still cause their accumulation, leading to cytotoxicity. This is counterproductive to tissue repair and may even induce hepatotoxicity and nephrotoxicity, ultimately endangering life [189]. Degradation of PLGA/PCL releases acidic by-products such as lactic acid and glycolic acid, which lower pH and trigger chronic inflammation. Additionally, the acidic environment directly damages cell membranes, inhibits the proliferation and differentiation of osteoblasts, and constitutes a high-risk factor for the recurrence of residual tumors [190,191]. When designing materials for clinical use, ensuring they are inherently safe is a must, especially for research aimed at real-world medical applications. This focus on safety is key to successfully moving from lab development to actual patient use, directly affecting how well treatments work and patients’ recovery outcomes. When evaluating the safety of materials made using bifunctional strategies, a comprehensive assessment covering 4 main areas is used. First, cytocompatibility checks make sure the material does not harm cells, allowing normal cell attachment, growth, and development into a specific cell type. Second, hemocompatibility analysis is essential to study how the material interacts with blood components, aiming to avoid issues like blood cell damage, abnormal clotting, or immune system overreaction. Third, monitoring in vivo organ toxicity involves regularly checking major organs (such as the liver, kidneys, and heart) after the material is implanted, to ensure no structural or functional damage occurs over time. Together, this thorough evaluation approach provides a solid scientific foundation for confirming the safety of bifunctional strategy materials and serves as an important reference for advancing their use in real clinical settings.

#### Cytocompatibility checks

Cytocompatibility is fundamental to biomedical materials, as it ensures harmonious interactions with surrounding cells. Without good cytocompatibility, materials may cause cell damage, inhibit growth, or trigger adverse inflammatory or immune responses—ultimately undermining their ability to support tissue repair, promote implant integration, or exert therapeutic functions. This makes it a core prerequisite for ensuring the safety and clinical effectiveness of any biomaterial. Such assessments typically involve coculturing either extracts derived from implants or the implants themselves with various functional cells closely related to bone repair processes. These cell types include human umbilical vein endothelial cells (HUVECs) responsible for angiogenesis, human bone marrow mesenchymal stem cells (hBMSCs), rat bone marrow mesenchymal stem cells (rBMSCs) with osteogenic potential, rat calvaria osteoblasts (ROBs), and mouse embryonic osteoblasts (MC3T3-E1) that directly participate in bone formation. Through these coculture models, key indicators such as cell morphology, viability, and functional activity are systematically evaluated to reflect cytocompatibility.

**Fig. 10.**
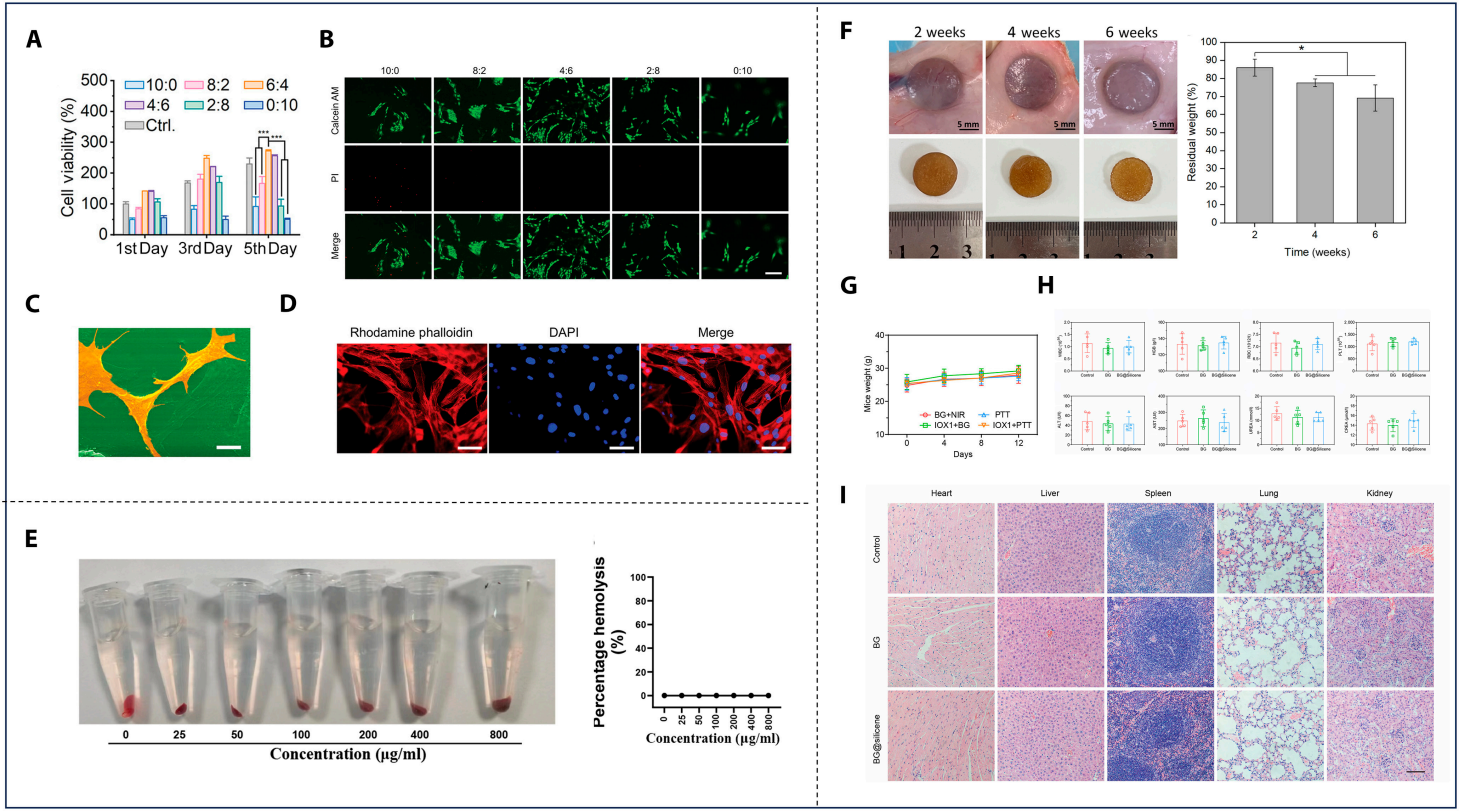
Material toxicity assessment of bifunctional strategic materials. (A) Cell viability of ROBs seeded on NG hydrogels20% after culturing for 1, 3, and 5 days. *n* = 4. ***P < 0.001. (B) Live/dead assay of ROBs seeded on different NG hydrogel (10:0, 8:2, 4:6, 2:8, and 0:10). (C) SEM image of ROBs seeded on 6:4 NG hydrogel. (D) Fluorescence images of β-actin protein and nucleus costained images of ROBs seeded on 6:4 NG hydrogel. Scale bar = 50 μm [70]. Copyright 2023, John Wiley and Sons. (E) Hemolysis assay of MCD with different concentrations and quantified results of hemolysis assay [138]. Copyright 2024, Elsevier. (F) 5NP/CMP@PAM hydrogel photos before and after removal from the skin and the residual weight of hydrogel after degradation [53]. Copyright 2022, Elsevier. (G) Time-dependent body weights of mice after BG@silicene scaffold treatments. (H) Hematological and biochemical indexes of the mice with the indicated treatments after 30 days. (I) H&E staining images of heart, liver, spleen, lung, and kidney obtained after mice were sacrificed [99]. Copyright 2023, Elsevier.

Virtually all bifunctional strategies undergo rigorous cytocompatibility verification during the design process, with numerous studies providing compelling evidence. For instance, with the previously mentioned ZIF-8/DOX/IDO printed scaffold [146], when rBMSCs were seeded onto the scaffold, the expression of cytoskeletal proteins (critical for cell adhesion and mechanical support) and the degree of cell spreading were higher than those in the negative control group, directly demonstrating the scaffold’s excellent cytocompatibility. In another study, Yao et al. [112] cocultured the extract of SOH1(CP)1 injectable hydrogel with BMSCs; by adjusting the incorporation amount of inorganic nanoparticles, they achieved optimal cytocompatibility that benefited hBMSC proliferation. Similarly, the MoS₂/NAGA/GelMA hydrogel [70] with MRI functionality validated the optimal NAGA/GelMA ratio for cell viability using CCK-8 and live/dead cell assays prior to osteogenic differentiation experiments (Fig. 10A and B), while scanning electron microscope (SEM) images and cytoskeleton staining showed ROBs growing and spreading in a spindle-like morphology with robust attachment (Fig. 10C and D).

#### Hemocompatibility

The hemolysis assay serves as a fundamental in vitro method for evaluating the biocompatibility and hematological safety of biomaterials, particularly those intended for use in blood-contacting applications such as implantable medical devices, drug delivery vehicles, and engineered nanomaterials. In this test, erythrocytes—commonly sourced from human donors or animal models such as rabbits or rats—are incubated with the test material or its extracts. The subsequent release of hemoglobin into the supernatant is quantitatively measured, providing an indicator of erythrocyte membrane disruption. A substantial level of hemolysis suggests membrane-destabilizing properties or cytotoxic effects, which may preclude clinical use. According to international standards including ISO 10993-4, a hemolysis ratio below 5% is generally considered acceptable, indicating that the material is nonhemolytic and compatible for biological applications [192]. This threshold helps ensure that materials introduced into the bloodstream do not induce acute or chronic damage to blood cells.

Building on these standardized evaluation protocols, the blood compatibility of 2 different biomaterial systems was assessed as examples. For the CS/nHA/CD scaffold [57], hemolysis testing revealed negligible erythrocyte damage, with the supernatant remaining optically clear and transparent after incubation with human red blood cells—visually indistinguishable from the phosphate buffered saline negative control. This stood in sharp contrast to the positive control group treated with deionized water, which exhibited a pronounced reddish hue indicative of substantial hemoglobin release from complete erythrocyte lysis. Extending these findings to nanoscale systems, concentration-dependent hemolysis assays of Cu₂S@BSA-loaded MSN/oDEX nanoparticles [138] similarly demonstrated minimal hemolytic effects across a broad concentration range (Fig. 10E). Quantitative analysis confirmed that both the macro-scaled CS/nHA/CD scaffold and nano-scaled MCD particles consistently exhibited hemolysis rates well below the 5% ISO 10993-4 threshold, affirming their potential for blood-contacting biomedical application.

#### In vivo toxicity

While the developed materials have demonstrated excellent biocompatibility and minimal cytotoxicity in standardized in vitro cell cultures, these findings cannot fully guarantee their absolute safety in biological systems. The physiological environment in vivo is substantially more complex than controlled laboratory conditions, involving dynamic interactions with immune components, metabolic processes, vascular systems, and neural networks—all of which may elicit unintended host responses not observed in cell-based assays. Furthermore, a comprehensive preclinical safety assessment must include careful examination of the material’s degradation kinetics, including the rate of degradation, the chemical composition of breakdown products, and how the body metabolizes and clears these residues to ensure that they are nontoxic and efficiently eliminated. Therefore, to fully evaluate the biosafety and functional performance of these biomaterials, rigorous in vivo studies using appropriate animal models are essential.

In simulated body fluid, the degradation behavior of the previously mentioned MoS₂/NAGA/GelMA hydrogel [70] was evaluated. Results indicated that the incorporation of Gel-MA enhanced the degradation capacity of the system. Specifically, for NG-20% and NG-30% hydrogels, an increase in the mass ratio of Gel-MA corresponded to an accelerated degradation rate. Although simulated body fluid provides a useful preliminary environment, it cannot fully replicate the complexities of the physiological conditions in living organisms. To better evaluate biodegradation in vivo, a subdermal implantation model in SD rats was employed for the 2.5NP/CMP@PAM hydrogel [53]. Observations indicated that the hydrogels transitioned from pure black to a lighter hue, suggesting progressive degradation of the embedded nanoparticles. After 6 weeks of implantation, the remaining weight of the hydrogel was 69.33% ± 7.27%, attributed largely to the slow degradation profile of CS (Fig. 10F).

In a related study, Liang et al. [99] conducted a subcutaneous implantation of BG and BG@silicene scaffolds in ICR mice maintained under specific pathogen-free conditions for 30 days. After the experimental period, the mice were euthanized, and comprehensive analyses were performed on blood and major organs. The results showed no statistically significant differences in body weight, hematological parameters, or blood biochemical markers between the experimental and control groups (Fig. 10G and H). Furthermore, histopathological examination using hematoxylin and eosin (H&E) staining revealed no observable damage or abnormalities in the main organs (Fig. 10I). Together, these hematological, biochemical, and histological results confirm that the BG and BG@silicene scaffolds exhibit excellent biocompatibility under in vivo conditions.

### Infection risk control

Postoperative infections are a serious concern after osteosarcoma surgery. The first and most important step to prevent this is to make sure that any implant placed in the body is completely sterile. This means using strict factory cleaning methods that kill all germs on the material without damaging its ability to function inside the body. Following international safety standards during manufacturing is essential to ensure every implant is safe before it is used. Common sterilization methods include thermal approaches (e.g., steam and dry heat), suitable for heat-resistant materials like metals, PEEK, and polyethylene; chemical methods such as ethylene oxide (EO) and hydrogen peroxide plasma, which are compatible with temperature-sensitive biodegradable polymers (e.g., PLGA and PCL) though residual toxicity and material oxidation must be considered; and radiation techniques (e.g., gamma and e-beam) that offer broad applicability but may cause degradation in certain polymers. The selection of an appropriate sterilization protocol must achieve a sterility assurance level of 10^−6^ while preserving the functional and structural properties of the implant material [193].

**Fig. 11.**
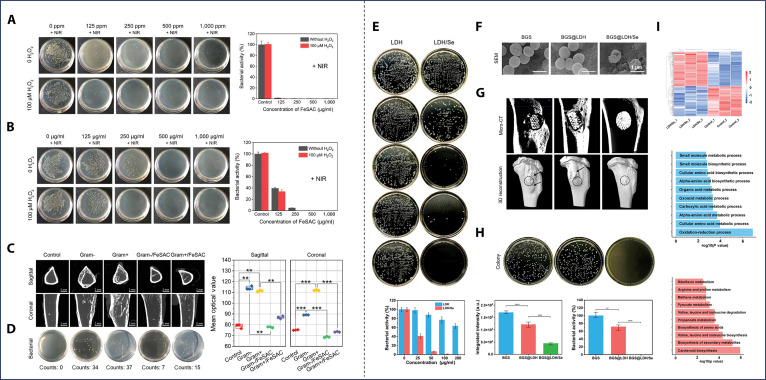
Infection risk control of bifunctional strategic materials. (A) Digital photographs of *S. aureus* after FeSAC coincubation with or without biological H_2_O_2_ and calculated bacterial activity. (B) Digital photographs and corresponding bacterial activity profile of the remaining *E. coli* inoculated agar plates after treatment with FeSAC suspension with NIR irradiation (808 nm, 1.5 W cm^−2^, 8 min) in the presence (100 × 10^−6^ m) or absence of H_2_O_2_. (C) Micro-CT scanning was performed on thigh bones harvested from SD rats 4 weeks after indicated treatments, with images presented in sagittal and coronal views, along with the corresponding mean optical values of the micro-CT images for different treatment groups in both views. (D) Digital photographs of agar plates and corresponding colony counting of the muscle tissues collected near the marrow cavity of different treatment groups [134]. Copyright 2021, John Wiley and Sons. (E) Digital images and quantitative analysis of bacterial colonies formed by MRSA incubated with LDH or LDH/Se at different concentrations. (F) SEM images of MRSA inoculated on BGS, BGS@LDH, and BGS@LDH/Se. (G) Micro-CT images and quantitative analysis of the severity of periprosthetic infection treated with indicated therapies in rabbit upper tibia. (H) Quantitative analysis of the bacterial colonies formed by the periarticular tissues from BGS, BGS@LDH, and BGS@LDH/Se groups. (I) A heatmap shows the differential gene expression pattern between the LDH/Se group and the control group, while GO and KEGG analyses enrich down-regulated genes into key biological processes [105]. Copyright 2024, John Wiley and Sons.

Beyond initial sterility, a second strategic direction involves engineering intrinsic antimicrobial functionality into the biomaterial to proactively defend against bacterial invasion during and after surgery [194]. Generally, therapeutic strategies designed to eliminate tumor cells, such as PTT and CDT, also exhibit potent antibacterial effects. The FeSAC/β-BG composite demonstrated strong antibacterial properties through a combination of nanocatalytic and photothermal mechanisms [134]. In vitro experiments showed that a concentration of 250 μg·ml^−1^ under NIR light irradiation completely eliminated both *Staphylococcus aureus* (Fig. 11A) and *Escherichia coli* within 4 h (Fig. 11B). In a rat osteomyelitis model, local administration of FeSAC suspension for 4 weeks markedly reduced infection levels and bacterial load, supported by micro-CT and bacteriological culture analyses (Fig. 11C and D). These results confirm the material’s efficacy in controlling complex bacterial infections in biological settings.

However, implant-associated infections involving drug-resistant bacteria further complicate treatment. The LDH/Se nanocomposite exhibited enhanced antibacterial performance, particularly against drug-resistant strains [105]. At 200 μg·ml^−1^, it achieved complete eradication of methicillin-resistant *S. aureus* (MRSA), outperforming LDH alone (Fig. 11E). SEM imaging revealed severe morphological damage, including cell shrinkage and membrane rupture in MRSA treated with LDH/Se (Fig. 11F). In a rabbit implant-associated infection model, BGS@LDH/Se scaffolds effectively suppressed infection without inducing pathological bone changes, and surrounding tissues showed no bacterial growth (Fig. 11G and H). Transcriptomic analysis indicated that the antibacterial activity originated from disruption of bacterial energy metabolism and redox homeostasis. Key mechanisms included inhibition of respiratory electron transport, adenosine triphosphate synthesis, amino acid metabolism, and carotenoid biosynthesis—compromising bacterial antioxidant defenses and membrane integrity (Fig. 11I).

## Summary and Perspectives

Postoperative osteosarcoma management has long been a clinical challenge, rooted in 2 intertwined issues: eradicating residual tumor cells to prevent recurrence and repairing critical-sized bone defects to restore limb function. Dual-function biomaterials, which integrate both antitumor and bone-regenerative capabilities into a single platform, have emerged as the most promising solution to this dilemma. To summary the field systematically, we compare 3 dual-function strategies, highlighting their strengths, weaknesses, and clinical alignment. Subsequently, we analyze basic research challenges and solutions, outline future trends for their development, and discuss clinical translation barriers and feasible resolutions.

### Systematic comparison of 3 dual-function strategies

Building on the earlier classification, this section provides a systematic comparison of the 3 primary bifunctional strategies, analyzing their design concepts, advantages, drawbacks, and consistency with clinical needs. Initially, researchers simply integrated antitumor materials with bone regeneration-promoting materials. However, this design was relatively simplistic and crude, neglecting the complexity of the postoperative microenvironment in osteosarcoma cases. Gradually, more sophisticated designs were introduced into composite materials: How to minimize damage to surrounding tissues during tumor elimination? How to reconstruct a favorable local microenvironment after tumor eradication? At the same time, osteosarcoma has a high degree of malignancy and a high chance of recurrence. Many bifunctional materials have also been designed to target stronger tumor killing effects in order to obtain the greatest benefit for patients. These strategies include enhancing tumor thermal sensitivity to thermogenic materials, enhancing generation efficiency for biomaterials that can produce ROS, the combined use of multiple antitumor modalities, and so on. With increasing understanding of the complex postoperative microenvironment in osteosarcoma, researchers recognize that eliminating residual tumors inevitably affects surrounding normal tissues, while premature tissue regeneration promotion may adversely influence tumor recurrence. Therefore, there is a growing interest in temporal regulatory strategies designed to eradicate tumors prior to promoting bone regeneration. The simplest strategy is to extend the time effect of contributing to bone function, while the time for antitumor duration is relatively short. However, the disadvantage of this strategy is that it still contributes to the release of bone components in the early antitumor stage and may adversely affect tumor killing. Another strategy is the core–shell structure, which achieves temporal regulation by first contacting the shell-antineoplastic structure and then contacting the nucleus-layer structure that promotes bone regeneration. At the same time, the time trajectory of antitumor and bone regeneration after osteosarcoma surgery can be better matched by regulating the degradation rate of the putamen–core structure. The third strategy is to give the biomaterial an “external remote-control switch”, which artificially controls to turn off the antitumor stage or turn on the pro-bone regeneration stage when we believe the antitumor stage is over. This strategy has more freedom to artificially control antitumor and pro-bone regeneration time points and is more conducive to individualized treatment of clinical patients. These 3 strategies together show the field’s progress from simple codelivery to advanced, clinically useful designs.

### Current challenges in basic research

Although bifunctional biomaterials hold substantial promise for post-osteosarcoma therapy, current experimental systems and design strategies face several critical challenges. Firstly, the discrepancy between experimental models and actual clinical scenarios needs to be addressed. Many studies have only verified the killing effect of materials on tumor cell lines in vitro, which ignores the intricate interactions and synergistic effects within the osteosarcoma microenvironment. The development of organoid technology offers hope for better studies of the osteosarcoma microenvironment, which provides highly biomimetic 3D disease models to mimic the cellular composition and spatial structure of native tissues [195,196]. This model successfully simulates the invasive growth pattern of osteosarcoma and shows unique advantages for studying the sensitivity of drugs loaded on materials and revealing the specific mechanism of the tumor microenvironment [197,198]. Simultaneously, many animal studies employ experimental designs that independently assess material anti-osteosarcoma efficacy and bone regeneration-promoting effects, failing to accurately replicate clinical scenarios. Future research should evaluate these dual functions in animal models better simulating post-osteosarcoma resection conditions, which raises another critical issue—the absence of standardized postoperative osteosarcoma models. Current research indicates substantial variations in tumor resection standards. Typically, an osteosarcoma model is first established, followed by tumor resection, with the resection range varying from the complete removal of visible tumors to the removal of 90% of the tumor mass [145,156,158]. We propose a novel conceptual approach to model establishment: first, develop a standardized bone defect model at sites such as the tibia, femur, or other skeletal regions in animals; next, quantitatively transplant tumor cells/tissues into the defect area; and subsequently administer interventions. This method enables precise control over the initial number of local residual tumor cells prior to intervention, potentially enhancing the simulation of post-resection scenarios for osteosarcoma while ensuring model consistency.

### Future prospects and challenges in clinical translation

**Fig. 12.**
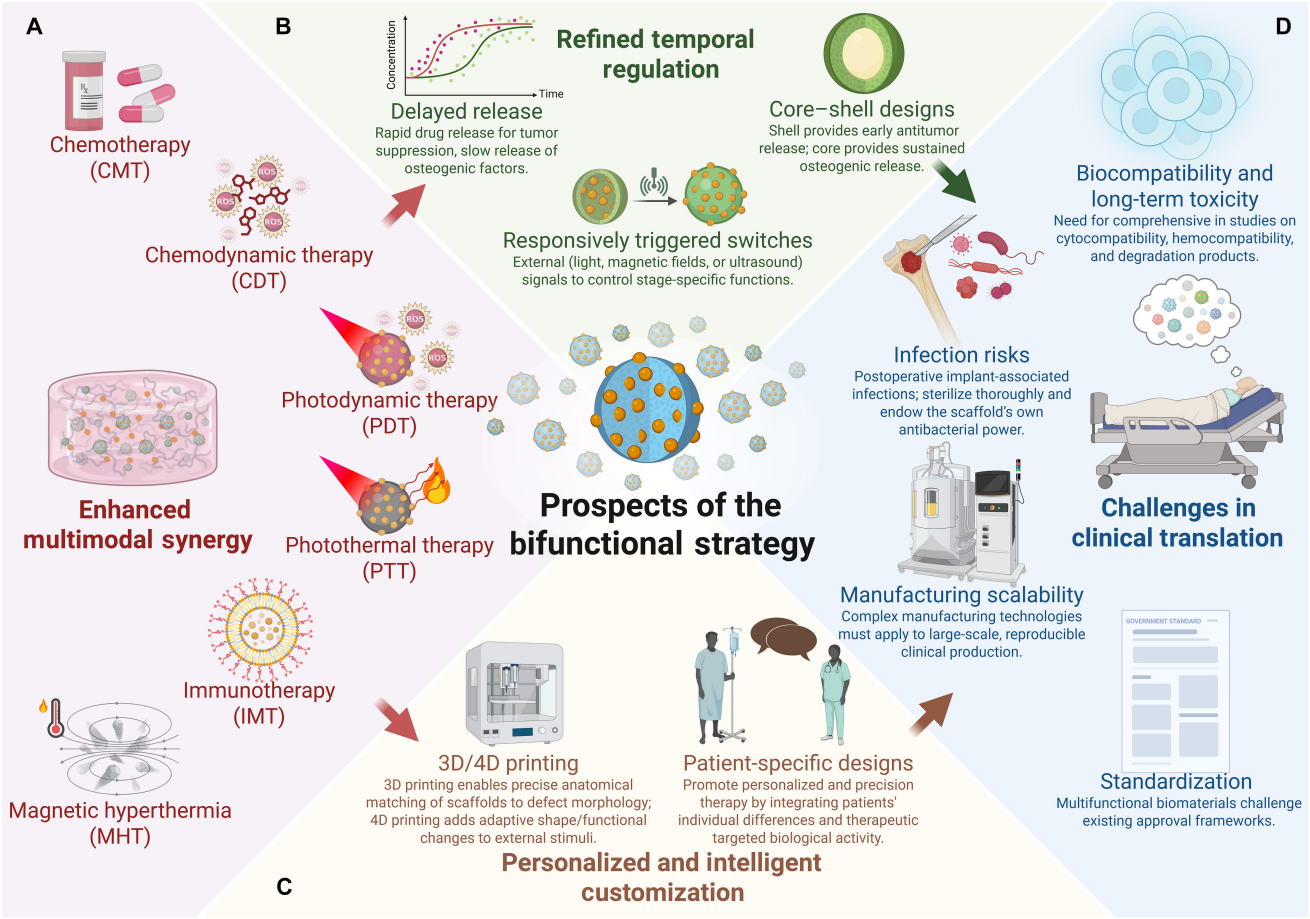
Future trends of bifunctional biomaterials in postoperative osteosarcoma therapy. (A) Multimodal synergy integrates therapies such as PTT, MHT, CMT, chemodynamic and photodynamic approaches, and immunotherapy into single platforms. By combining complementary mechanisms—for example, PTT with CMT to enhance drug penetration and osteogenic ion release, or MHT with CDT to boost ROS—these systems maximize tumor suppression while promoting bone regeneration. (B) Temporal regulation addresses the conflict between tumor control and bone healing. Strategies such as sequential release profiles, core–shell architectures, and externally triggered switches (light, magnetic fields, and ultrasound) ensure early elimination of residual tumor cells followed by delayed osteogenesis, reducing recurrence risk and enhancing functional repair. (C) Personalized customization leverages 3D and 4D printing to fabricate scaffolds tailored to patient-specific defect geometries, with 4D systems adding dynamic shape or functional adaptation. These implants can be coupled with imaging-guided design and individualized drug release, aligning treatment with patient needs. (D) Substantial clinical translation challenges remain, including long-term safety and degradation toxicity, infection risk, manufacturing scalability, regulatory approval, and high costs. Overcoming these barriers will be essential for advancing dual-functional biomaterials from promising experimental concepts to reliable clinical solutions.

As mentioned above, dual-function strategies are gradually evolving toward 2 directions: those that enhance antitumor efficacy (Fig. 12A) and temporal strategies (Fig. 12B). Meanwhile, personalized therapy is also gradually attracting increasing attention (Fig. 12C). Unlike one-size-fits-all conventional scaffolds, personalized strategies begin with structural tailoring: 3D printing uses patient-specific imaging data to create scaffolds that exactly fit the unique shape and size of irregular bone defects, eliminating gaps where residual tumors might lurk. 4D printing takes this further by adding dynamic adaptability, enabling scaffolds to adjust their structure or function over time to match the patient’s treatment stages (e.g., shifting from tumor elimination to bone regeneration). Enhancing targeting precision is another core component of personalized therapy. Biomaterials can boost this precision through multiple mechanisms: first is biomarker-mediated active targeting, which binds specifically to molecules overexpressed on osteosarcoma cells; second is passive targeting—a phenomenon where macromolecules accumulate selectively and remain in tumor tissues for an extended period, all thanks to the unique structure of tumor blood vessels; third is tumor microenvironment responsiveness, which allows therapeutic agents to be released on demand in the tumor’s distinct physicochemical environment; and fourth is externally triggered regulation, which modulates the timing and intensity of treatment using physical stimuli. The integration of personalized structural carriers and these targeted pathways not only solves the problem of incomplete tumor coverage with conventional scaffolds but also mitigates the off-target toxicity of traditional treatments. This makes it a key research priority for moving biomaterials from preclinical studies to clinical use.

The practical translation of biomaterials from laboratory to clinical practice still faces numerous challenges (Fig. 12D). Although toxicity evaluations have confirmed the biosafety of these materials under laboratory conditions, safety assessment and long-term efficacy monitoring during clinical translation remain indispensable steps that cannot be overlooked. This process requires interdisciplinary collaboration—bringing together materials science, oncology, orthopedics, and toxicology—to address potential issues such as immunogenicity, accumulation of metabolic by-products, and dynamic changes at the tissue–material interface during long-term implantation. Sterilization of implanted materials also poses a critical challenge. Common sterilization methods may impair the bioactivity or functional performance of biomaterials. Balancing effective sterilization with the preservation of material functionality remains an unresolved issue that demands further investigation. Additionally, preclinical antibacterial studies of implanted materials typically use single bacterial strains, yet clinical infections are often polymicrobial. This discrepancy means the actual antibacterial efficacy of materials in clinical settings may be lower than observed in preclinical tests, requiring more realistic polymicrobial models to validate antibacterial performance. Another pressing challenge lies in scalability and manufacturing. Consistently reproducing material properties on a large scale remains difficult, especially for complex systems integrating nanoparticles, growth factors, and controlled-release mechanisms. Few studies address industrial-scale production requirements, such as batch-to-batch uniformity, long-term storage stability, and cost-effectiveness—critical factors for commercialization. Moreover, the lack of universally accepted critical quality attributes for bifunctional biomaterials further complicates technology transfer from research laboratories to manufacturing facilities. A potential solution to these manufacturing challenges is the adoption of a modular material design strategy. By developing standardized, prevalidated functional components—such as drug-loaded modules with well-defined release kinetics or osteogenic modules with confirmed bioactivity—researchers and manufacturers can assemble tailored biomaterial systems that are easier to produce, qualify, and regulate. This approach not only enhances reproducibility but also streamlines clinical adoption by reducing production complexity and quality variability. Finally, it is essential to establish and refine standards for bifunctional biomaterials. Clear guidelines covering performance benchmarks, safety thresholds, and clinical evaluation protocols will provide more effective guidance for their clinical application.

## Conclusion

Dual-function biomaterials is a vital approach for postoperative osteosarcoma management, addressing the key needs of this clinical challenge. Looking ahead, their development will keep advancing toward more synergistic, clinically adapted directions—prioritizing better alignment with treatment goals and individual patient needs. While there are still hurdles in translating these materials from research to clinical practice, these can be addressed through interdisciplinary collaboration and optimized design and ultimately advancing them to routine clinical use.

## References

[1] Jafari F, Javdansirat S, Sanaie S, Naseri A, Shamekh A, Rostamzadeh D, Dolati S. Osteosarcoma: A comprehensive review of management and treatment strategies. Ann Diagn Pathol. 2020;49: Article 151654. 33130384 10.1016/j.anndiagpath.2020.151654

[2] Beird HC, Bielack SS, Flanagan AM, Gill J, Heymann D, Janeway KA, Livingston JA, Roberts RD, Strauss SJ, Gorlick R. Osteosarcoma. Nat Rev Dis Primers. 2022;8(1):77. 36481668 10.1038/s41572-022-00409-y

[3] Panez-Toro I, Munoz-Garcia J, Vargas-Franco JW, Renodon-Corniere A, Heymann MF, Lezot F, Heymann D. Advances in osteosarcoma. Curr Osteoporos Rep. 2023;21(4):330–343. 37329384 10.1007/s11914-023-00803-9 PMC10393907

[4] Zeng F, Li C, Wang H, Wang Y, Ren T, He F, Jiang J, Xu J, Wang B, Wu Y, et al. Intraoperative resection guidance and rapid pathological diagnosis of osteosarcoma using B7H3 targeted probe under NIR-II fluorescence imaging. Adv Sci. 2024;11(33): Article e2310167. 10.1002/advs.202310167 PMC11434027 38502871

[5] Lu Y, Zhang J, Chen Y, Kang Y, Liao Z, He Y, Zhang C. Novel immunotherapies for osteosarcoma. Front Oncol. 2022;12: Article 830546. 35433427 10.3389/fonc.2022.830546 PMC9012135

[6] Shi P, Cheng Z, Zhao K, Chen Y, Zhang A, Gan W, Zhang Y. Active targeting schemes for nano-drug delivery systems in osteosarcoma therapeutics. J Nanobiotechnology. 2023;21(1):103. 36944946 10.1186/s12951-023-01826-1 PMC10031984

[7] Holmboe L, Andersen AM, Morkrid L, Slordal L, Hall KS. High dose methotrexate chemotherapy: Pharmacokinetics, folate and toxicity in osteosarcoma patients. Br J Clin Pharmacol. 2012;73(1):106–114. 21707700 10.1111/j.1365-2125.2011.04054.x PMC3248260

[8] Pugazhendhi A, Edison T, Velmurugan BK, Jacob JA, Karuppusamy I. Toxicity of doxorubicin (dox) to different experimental organ systems. Life Sci. 2018;200:26–30. 29534993 10.1016/j.lfs.2018.03.023

[9] Wen Y, Tang F, Tu CQ, Hornicek F, Duan ZF, Min L. Immune checkpoints in osteosarcoma: Recent advances and therapeutic potential. Cancer Lett. 2022;547: Article 215887. 35995141 10.1016/j.canlet.2022.215887

[10] Tawbi HA, Burgess M, Bolejack V, Van Tine BA, Schuetze SM, Hu J, D’angelo S, Attia S, Riedel RF, Priebat DA, et al. Pembrolizumab in advanced soft-tissue sarcoma and bone sarcoma (SARC028): A multicentre, two-cohort, single-arm, open-label, phase 2 trial. Lancet Oncol. 2017;18(11):1493–1501. 28988646 10.1016/S1470-2045(17)30624-1 PMC7939029

[11] Carina V, Costa V, Sartori M, Bellavia D, De Luca A, Raimondi L, Fini M, Giavaresi G. Adjuvant biophysical therapies in osteosarcoma. Cancer. 2019;11(3): Article 348. 10.3390/cancers11030348 PMC6468347 30871044

[12] Zhao LP, Zhang X, Wang XX, Guan XW, Zhang WF, Ma JL. Recent advances in selective photothermal therapy of tumor. J Nanobiotechnology. 2021;19(1): Article 335. 34689765 10.1186/s12951-021-01080-3 PMC8543909

[13] Xie M, Gong T, Wang Y, Li Z, Lu M, Luo Y, Min L, Tu C, Zhang X, Zeng Q, et al. Advancements in photothermal therapy using near-infrared light for bone tumors. Int J Mol Sci. 2024;25(8): Article 4139. 38673726 10.3390/ijms25084139 PMC11050412

[14] Li X, Zhang R, Yang Y, Huang W. Finely tailored conjugated small molecular nanoparticles for near-infrared biomedical applications. Research. 2025;8: Article 0534. 39801503 10.34133/research.0534 PMC11717998

[15] Shi Z, Bai H, Wu J, Miao X, Gao J, Xu X, Liu Y, Jiang J, Yang J, Zhang J, et al. Acceptor engineering produces ultrafast nonradiative decay in NIR-II aza-BODIPY nanoparticles for efficient osteosarcoma photothermal therapy via concurrent apoptosis and pyroptosis. Research. 2023;6: Article 0169. 37342631 10.34133/research.0169 PMC10278946

[16] Wang J, Zhou J, Xie Z, Zhang Y, He M, Wei T, Wu S, Du C. Multifunctional 4D printed shape memory composite scaffolds with photothermal and magnetothermal effects for multimodal tumor therapy and bone repair. Biofabrication. 2025;17(2): Article 025032. 10.1088/1758-5090/adc29e 40106897

[17] Chen X, Tan LF, Liu TL, Meng XW. Micro-nanomaterials for tumor microwave hyperthermia design preparation, and application. Curr Drug Deliv. 2017;14(3):307–322. 26743355 10.2174/1567201813666160108113805

[18] Gao H, Cao Z, Liu H, Chen L, Bai Y, Wu Q, Yu X, Wei W, Wang M. Multifunctional nanomedicines-enabled chemodynamic-synergized multimodal tumor therapy via Fenton and Fenton-like reactions. Theranostics. 2023;13(6):1974–2014. 37064867 10.7150/thno.80887 PMC10091877

[19] Zhang Y, Feng G, He T, Yang M, Lin J, Huang P. Traceable lactate-fueled self-acting photodynamic therapy against triple-negative breast cancer. Research. 2024;7: Article 0277. 40771576 10.34133/research.0277 PMC12326368

[20] Zhang D, Tan J, Xu R, Du H, Xie J, Peng F, Liu X. Collaborative design of MgO/FeO_x_ nanosheets on titanium: Combining therapy with regeneration. Small. 2023;19(5): Article e2204852. 36464630 10.1002/smll.202204852

[21] Fang H, Zhu D, Chen Y, Zhang C, Li G, Fang Q, Chang M, Chen Y, Gao Y. Ultrasound-responsive 4D bioscaffold for synergistic sonopiezoelectric-gaseous osteosarcoma therapy and enhanced bone regeneration. Adv Sci. 2025;12(22): Article 2417208. 10.1002/advs.202417208 PMC12165076 40178027

[22] Xie C, Ye JC, Liang RJ, Yao XD, Wu XY, Koh YW, Wei W, Zhang XZ, Ouyang HW. Advanced strategies of biomimetic tissue-engineered grafts for bone regeneration. Adv Healthc Mater. 2021;10(14): Article 2100408. 10.1002/adhm.202100408 33949147

[23] Niu X, Xiao S, Huang R, Huang D, Aifantis KE, Yu H, Xue C, Yin L, Dunne N, Li X. ZIF-8-modified hydrogel sequentially delivers angiogenic and osteogenic growth factors to accelerate vascularized bone regeneration. J Control Release. 2024;374:154–170. 39127448 10.1016/j.jconrel.2024.08.011

[24] Zhang Z, Bao L, Qian C, Furtado M, Li H, Guo S, Zheng Y, Fu D, Dong K, Cui W, et al. The high-strength and toughness Janus bionic periosteum matching bone development and growth in children. Compos Part B Eng. 2023;256: Article 110642.

[25] Wang B, Xiao S, Liao J, Huang Y, Guan X, Wang C, Xue C, Cai Q, Li X. Directional biomimetic scaffold-mediated cell migration and pathological microenvironment regulation accelerate diabetic bone defect repair. ACS Nano. 2025;19(36):32382–32404. 40919889 10.1021/acsnano.5c08238

[26] Xu Y, Xu C, He L, Zhou JJ, Chen TW, Ouyang L, Guo XD, Qu YZ, Luo ZQ, Duan DY. Stratified-structural hydrogel incorporated with magnesium-ion-modified black phosphorus nanosheets for promoting neuro-vascularized bone regeneration. Bioact Mater. 2022;16:271–284. 35386320 10.1016/j.bioactmat.2022.02.024 PMC8965728

[27] Niu XL, Xiao SZ, Li KM, He W, Huang D, Yu H, Xue C, Cai Q, Dunne N, Li XM. Nanofiber-reinforced self-adaptive hydrogels with desired sequential delivery capability of bioactive factors and magnesium for vascularized osteochondral regeneration. Adv Funct Mater. 2025; Article 2503948.

[28] Xu YR, Saiding Q, Zhou X, Wang J, Cui WG, Chen XL. Electrospun fiber-based immune engineering in regenerative medicine. Smart Med. 2024;3(1): Article e20230034. 39188511 10.1002/SMMD.20230034 PMC11235953

[29] Wang F, Ye Y, Zhang Z, Teng W, Sun H, Chai X, Zhou X, Chen J, Mou H, Eloy Y, et al. PDGFR in PDGF-BB/PDGFR signaling pathway does orchestrates osteogenesis in a temporal manner. Research. 2023;6: Article 0086. 37223474 10.34133/research.0086 PMC10202377

[30] Autelitano L, Meazzini MC. Alveolar cleft reconstruction with vomerine bone: Two surgical procedures in one step: A case series. Plast Aesthet Res. 2023;10: Article 16.

[31] Dimitriou R, Jones E, Mcgonagle D, Giannoudis PV. Bone regeneration: Current concepts and future directions. BMC Med. 2011;9(1): Article 66. 21627784 10.1186/1741-7015-9-66 PMC3123714

[32] Dai M, Lin X, Hua P, Wang S, Sun X, Tang C, Zhang C, Liu L. Antibacterial sequential growth factor delivery from alginate/gelatin methacryloyl microspheres for bone regeneration. Int J Biol Macromol. 2024;275(Pt 1): Article 133557. 38955293 10.1016/j.ijbiomac.2024.133557

[33] Tang X, Wang Y, Liu N, Deng X, Zhou Z, Yu C, Wang Y, Fang K, Wu T. Methacrylated carboxymethyl chitosan scaffold containing icariin-loaded short fibers for antibacterial, hemostasis, and bone regeneration. ACS Biomater Sci Eng. 2024;10(8):5181–5193. 38935742 10.1021/acsbiomaterials.4c00707

[34] Nie R, Zhang QY, Feng ZY, Huang K, Zou CY, Fan MH, Zhang YQ, Zhang JY, Li-Ling J, Tan B, et al. Hydrogel-based immunoregulation of macrophages for tissue repair and regeneration. Int J Biol Macromol. 2024;268(Pt 1): Article 131643. 38643918 10.1016/j.ijbiomac.2024.131643

[35] Fan L, Chen S, Yang M, Liu Y, Liu J. Metallic materials for bone repair. Adv Healthc Mater. 2024;13(3): Article e2302132. 37883735 10.1002/adhm.202302132

[36] Šalandová M, van Hengel IA, Apachitei I, Zadpoor AA, van der Eerden BC, Fratila-Apachitei LE. Inorganic agents for enhanced angiogenesis of orthopedic biomaterials. Adv Healthc Mater. 2021;10(12): Article 2002254. 34036754 10.1002/adhm.202002254 PMC11469191

[37] Gu KJ, Tan Y, Li ST, Chen SY, Lin KL, Tang YM, Zhu M. Sensory nerve regulation via H3K27 demethylation revealed in Akermanite composite microspheres repairing maxillofacial bone defect. Adv Sci. 2024;11(30): Article 2400242. 10.1002/advs.202400242 PMC11321702 38874525

[38] Song WC, Zhao DL, Wang JJ, Han ZS, Liu YJ, Wang YF, Yang C. Ultrasound-driven innervated bone regeneration in additively manufactured degradable metallic scaffolds. Adv Healthc Mater. 2025;14(12): Article 2404024. 10.1002/adhm.202404024 40152173

[39] Filip DG, Surdu VA, Paduraru AV, Andronescu E. Current development in biomaterials—Hydroxyapatite and bioglass for applications in biomedical field: A review. J Funct Biomater. 2022;13(4): Article 248. 36412889 10.3390/jfb13040248 PMC9680477

[40] Zhang Y, He F, Zhang Q, Lu H, Yan S, Shi X. 3D-printed flat-bone-mimetic bioceramic scaffolds for cranial restoration. Research. 2023;6: Article 0255. 37899773 10.34133/research.0255 PMC10603392

[41] Bai YX, Wang ZJ, He XL, Zhu YJ, Xu X, Yang HY, Mei GY, Chen SG, Ma B, Zhu RR. Application of bioactive materials for osteogenic function in bone tissue engineering. Small Methods. 2024;8(8): Article 2301283. 10.1002/smtd.202301283 38509851

[42] Li J, Ma J, Feng Q, Xie E, Meng Q, Shu W, Wu J, Bian L, Han F, Li B. Building osteogenic microenvironments with a double-network composite hydrogel for bone repair. Research. 2023;6: Article 0021. 37040486 10.34133/research.0021 PMC10076009

[43] Zhao Z, Ruan H, Chen A, Xiong W, Zhang M, Cai M, Cui W. Genetic engineered ultrasound-triggered injectable hydrogels for promoting bone reconstruction. Research. 2023;6: Article 0221. 39830009 10.34133/research.0221 PMC11740919

[44] Shin J, Kang NY, Kim B, Hong HYS, Yu L, Kim J, Kang HM, Kim JS. One-dimensional nanomaterials for cancer therapy and diagnosis. Chem Soc Rev. 2023;52(13):4488–4514. 37338931 10.1039/d2cs00840h

[45] Yuan P, Min Y, Zhao Z. Multifunctional nanoparticles for the treatment and diagnosis of osteosarcoma. Biomater Adv. 2023;151: Article 213466. 37229927 10.1016/j.bioadv.2023.213466

[46] Li Y, Liu C. Nanomaterial-based bone regeneration. Nanoscale. 2017;9(15):4862–4874. 28358401 10.1039/c7nr00835j

[47] Chen W, Zhang H, Zhou Q, Zhou F, Zhang Q, Su J. Smart hydrogels for bone reconstruction via modulating the microenvironment. Research. 2023;6: Article 0089. 36996343 10.34133/research.0089 PMC10042443

[48] Tang Z, Deng L, Zhang J, Jiang T, Xiang H, Chen Y, Liu H, Cai Z, Cui W, Xiong Y. Intelligent hydrogel-assisted hepatocellular carcinoma therapy. Research. 2024;7: Article 0477. 39691767 10.34133/research.0477 PMC11651419

[49] Wang Y, Min L, Lu M, Zhou Y, Wang J, Zhang Y, Yu X, Tang F, Luo Y, Duan H, et al. The functional outcomes and complications of different reconstruction methods for giant cell tumor of the distal radius: Comparison of osteoarticular allograft and three-dimensional-printed prosthesis. BMC Musculoskelet Disord. 2020;21(1): Article 69. 32013950 10.1186/s12891-020-3084-0 PMC6998256

[50] Cui X, Ruan Q, Zhuo X, Xia X, Hu J, Fu R, Li Y, Wang J, Xu H. Photothermal nanomaterials: A powerful light-to-heat converter. Chem Rev. 2023;123(11):6891–6952. 37133878 10.1021/acs.chemrev.3c00159 PMC10273250

[51] Wang Z, Zhao J, Tang W, Hu L, Chen X, Su Y, Zou C, Wang J, Lu WW, Zhen W, et al. Multifunctional nanoengineered hydrogels consisting of black phosphorus nanosheets upregulate bone formation. Small. 2019;15(41): Article e1901560. 31423735 10.1002/smll.201901560

[52] Bigham A, Fasolino I, Borsacchi S, Valente C, Calucci L, Turacchio G, Pannico M, Serrano-Ruiz M, Ambrosio L, Raucci MG. A theragenerative bio-nanocomposite consisting of black phosphorus quantum dots for bone cancer therapy and regeneration. Bioact Mater. 2024;35:99–121. 38283385 10.1016/j.bioactmat.2024.01.018 PMC10818087

[53] Li C, Zhang W, Wang R, Du X-F, Jiang D, Liu B, Nie Y, Liao J, Chen Y, Liang X, et al. Nanocomposite multifunctional hydrogel for suppressing osteosarcoma recurrence and enhancing bone regeneration. Chem Eng J. 2022;435: Article 134896.

[54] Dang W, Ma B, Li B, Huan Z, Ma N, Zhu H, Chang J, Xiao Y, Wu C. 3D printing of metal-organic framework nanosheets-structured scaffolds with tumor therapy and bone construction. Biofabrication. 2020;12(2): Article 025005. 31756727 10.1088/1758-5090/ab5ae3

[55] Dai W, Zheng Y, Li B, Yang F, Chen W, Li Y, Deng Y, Bai D, Shu R. A 3D-printed orthopedic implant with dual-effect synergy based on MoS_2_ and hydroxyapatite nanoparticles for tumor therapy and bone regeneration. Colloids Surf B Biointerfaces. 2023;228: Article 113384. 37320980 10.1016/j.colsurfb.2023.113384

[56] Yang B, Yin J, Chen Y, Pan S, Yao H, Gao Y, Shi J. 2D-black-phosphorus-reinforced 3D-printed scaffolds: A stepwise countermeasure for osteosarcoma. Adv Mater. 2018;30(10): Article 1705611. 10.1002/adma.201705611 29333689

[57] Lu Y, Li L, Li M, Lin Z, Wang L, Zhang Y, Yin Q, Xia H, Han G. Zero-dimensional carbon dots enhance bone regeneration, osteosarcoma ablation, and clinical bacterial eradication. Bioconjug Chem. 2018;29(9):2982–2993. 29986578 10.1021/acs.bioconjchem.8b00400 PMC6380686

[58] Yang C, Ma H, Wang Z, Younis MR, Liu C, Wu C, Luo Y, Huang P. 3D printed wesselsite nanosheets functionalized scaffold facilitates NIR-II photothermal therapy and vascularized bone regeneration. Adv Sci. 2021;8(20): Article e2100894. 10.1002/advs.202100894 PMC8529444 34396718

[59] Pan S, Yin J, Yu L, Zhang C, Zhu Y, Gao Y, Chen Y. 2D MXene-integrated 3D-printing scaffolds for augmented osteosarcoma phototherapy and accelerated tissue reconstruction. Adv Sci. 2020;7(2): Article 1901511. 10.1002/advs.201901511 PMC6974945 31993282

[60] Yin J, Pan S, Guo X, Gao Y, Zhu D, Yang Q, Gao J, Zhang C, Chen Y. Nb_2_C MXene-functionalized scaffolds enables osteosarcoma phototherapy and angiogenesis/osteogenesis of bone defects. Nanomicro Lett. 2021;13(1):30. 34138204 10.1007/s40820-020-00547-6 PMC8187678

[61] He C, Dong C, Yu L, Chen Y, Hao Y. Ultrathin 2D inorganic ancient pigment decorated 3D-printing scaffold enables photonic hyperthermia of osteosarcoma in NIR-II biowindow and concurrently augments bone regeneration. Adv Sci. 2021;8(19): Article e2101739. 10.1002/advs.202101739 PMC8498872 34338444

[62] Long J, Zhang W, Chen Y, Teng B, Liu B, Li H, Yao Z, Wang D, Li L, Yu XF, et al. Multifunctional magnesium incorporated scaffolds by 3D-printing for comprehensive postsurgical management of osteosarcoma. Biomaterials. 2021;275:120950. 34119886 10.1016/j.biomaterials.2021.120950

[63] Wang L, Yuan L, Dong Y, Huang W, Zhu J, Du X, Zhang C, Liu P, Mo J, Li B, et al. Multifunctional 3D matrixes based on flexible bioglass nanofibers for potential application in postoperative therapy of osteosarcoma. Regen Biomater. 2024;11: Article rbae088. 39165883 10.1093/rb/rbae088 PMC11333569

[64] Du J, Ding H, Fu S, Li D, Yu B. Bismuth-coated 80S15C bioactive glass scaffolds for photothermal antitumor therapy and bone regeneration. Front Bioeng Biotechnol. 2022;10:1098923. 36760751 10.3389/fbioe.2022.1098923 PMC9907359

[65] Lu L, Wang H, Yang M, Wang L, Gan K. Three-dimensional-printed MPBI@beta-TCP scaffold promotes bone regeneration and impedes osteosarcoma under near-infrared laser irradiation. FASEB J. 2023;37(5): Article e22924. 37071462 10.1096/fj.202201991R

[66] Dong S, Zhang YN, Wan J, Cui R, Yu X, Zhao G, Lin K. A novel multifunctional carbon aerogel-coated platform for osteosarcoma therapy and enhanced bone regeneration. J Mater Chem B. 2020;8(3):368–379. 31782474 10.1039/c9tb02383f

[67] Zhang W, Gu J, Li K, Zhao J, Ma H, Wu C, Zhang C, Xie Y, Yang F, Zheng X. A hydrogenated black TiO_2_ coating with excellent effects for photothermal therapy of bone tumor and bone regeneration. Mater Sci Eng C Mater Biol Appl. 2019;102:458–470. 31147017 10.1016/j.msec.2019.04.025

[68] Cai B, Huang L, Wang J, Sun D, Zhu C, Huang Y, Li S, Guo Z, Liu L, Feng G, et al. 3D printed multifunctional Ti_6_Al_4_V-based hybrid scaffold for the management of osteosarcoma. Bioconjug Chem. 2021;32(10):2184–2194. 34491734 10.1021/acs.bioconjchem.1c00367

[69] Blomqvist L, Nordberg GF, Nurchi VM, Aaseth JO. Gadolinium in medical imaging—Usefulness, toxic reactions and possible countermeasures—A review. Biomolecules. 2022;12(6). 10.3390/biom12060742 PMC9221011 35740867

[70] Huang Y, Zhai X, Ma T, Zhang M, Yang H, Zhang S, Wang J, Liu W, Jin X, Lu WW, et al. A unified therapeutic-prophylactic tissue-engineering scaffold demonstrated to prevent tumor recurrence and overcoming infection toward bone remodeling. Adv Mater. 2023;35(25): Article e2300313. 36939167 10.1002/adma.202300313

[71] Liu Y, Fan H, Guo Q, Jiang A, Du X, Zhou J. Ultra-small pH-responsive Nd-doped NaDyF_4_ nanoagents for enhanced cancer theranostic by in situ aggregation. Theranostics. 2017;7(17):4217–4228. 29158821 10.7150/thno.21557 PMC5695008

[72] Heng C, Zheng X, Hui J, Ma X, Fan D. Neodymium and manganese ions co-doped whitlockite for temperature monitoring, photothermal therapy, and bone tissue repair in osteosarcoma. J Colloid Interface Sci. 2024;653(Pt B):1488–1503. 37804617 10.1016/j.jcis.2023.09.186

[73] Melamed JR, Edelstein RS, Day ES. Elucidating the fundamental mechanisms of cell death triggered by photothermal therapy. ACS Nano. 2015;9(1):6–11. 25590560 10.1021/acsnano.5b00021

[74] Ma L, Zhou Y, Zhang Z, Liu Y, Zhai D, Zhuang H, Li Q, Yuye J, Wu C, Chang J. Multifunctional bioactive Nd-ca-si glasses for fluorescence thermometry, photothermal therapy, and burn tissue repair. Sci Adv. 2020;6(32): Article eabb1311. 32821831 10.1126/sciadv.abb1311 PMC7413731

[75] Zhou J, Zhao W, Miao Z, Wang J, Ma Y, Wu H, Sun T, Qian H, Zha Z. Folin–Ciocalteu assay inspired polyoxometalate nanoclusters as a renal clearable agent for non-inflammatory photothermal cancer therapy. ACS Nano. 2020;14(2):2126–2136. 32027121 10.1021/acsnano.9b08894

[76] Wang S-B, Zhang C, Chen Z-X, Ye J-J, Peng S-Y, Rong L, Liu C-J, Zhang X-Z. A versatile carbon monoxide nanogenerator for enhanced tumor therapy and anti-inflammation. ACS Nano. 2019;13(5):5523–5532. 31046229 10.1021/acsnano.9b00345

[77] Guedes G, Wang S, Fontana F, Figueiredo P, Linden J, Correia A, Pinto RJB, Hietala S, Sousa FL, Santos HA. Dual-crosslinked dynamic hydrogel incorporating {Mo_154_} with pH and NIR responsiveness for chemo-photothermal therapy. Adv Mater. 2021;33(40): Article e2007761. 34382257 10.1002/adma.202007761 PMC11468987

[78] Lu H, Li Z, Duan Z, Liao Y, Liu K, Zhang Y, Fan L, Xu T, Yang D, Wang S, et al. Photothermal catalytic reduction and bone tissue engineering towards a three-in-one therapy strategy for osteosarcoma. Adv Mater. 2024;36(40): Article e2408016. 39165073 10.1002/adma.202408016

[79] Yuan X, Kang Y, Li RY, Niu GL, Shi JC, Yang YW, Fan YY, Ye JM, Han JW, Pei ZC, et al. Magnetically triggered thermoelectric heterojunctions with an efficient magnetic-thermo-electric energy cascade conversion for synergistic cancer therapy. Nat Commun. 2025;16(1):2369. 40064895 10.1038/s41467-025-57672-2 PMC11894112

[80] Shi Y, Wang Z, Zhou X, Lin C, Chen C, Gao B, Xu W, Zheng X, Wu T, Wang H. Preparation of a 3D printable high-performance GelMA hydrogel loading with magnetic cobalt ferrite nanoparticles. Front Bioeng Biotechnol. 2023;11:1132192. 36937750 10.3389/fbioe.2023.1132192 PMC10017762

[81] Tavares F, Soares PIP, Silva JC, Borges JP. Preparation and in vitro characterization of magnetic CS/PVA/HA/pSPIONs scaffolds for magnetic hyperthermia and bone regeneration. Int J Mol Sci. 2023;24(2):1128. 36674644 10.3390/ijms24021128 PMC9863008

[82] Zhang Y, Zhai D, Xu M, Yao Q, Chang J, Wu C. 3D-printed bioceramic scaffolds with a Fe(3)O(4)/graphene oxide nanocomposite interface for hyperthermia therapy of bone tumor cells. J Mater Chem B. 2016;4(17):2874–2886. 32262965 10.1039/c6tb00390g

[83] Sreeja S, Parameshwar R, Varma PRH, Sailaja GS. Hierarchically porous osteoinductive poly(hydroxyethyl methacrylate-co-methyl methacrylate) scaffold with sustained doxorubicin delivery for consolidated osteosarcoma treatment and bone defect repair. ACS Biomater Sci Eng. 2021;7(2):701–717. 33395260 10.1021/acsbiomaterials.0c01628

[84] Lanzillotti C, Iaquinta MR, De Pace R, Mosaico M, Patergnani S, Giorgi C, Tavoni M, Dapporto M, Sprio S, Tampieri A, et al. Osteosarcoma cell death induced by innovative scaffolds doped with chemotherapeutics. J Cell Physiol. 2024;239(5): Article e31256. 38591855 10.1002/jcp.31256

[85] Jin H, Ji Y, Cui Y, Xu L, Liu H, Wang J. Simvastatin-incorporated drug delivery systems for bone regeneration. ACS Biomater Sci Eng. 2021;7(6):2177–2191. 33877804 10.1021/acsbiomaterials.1c00462

[86] Dehghankelishadi P, Maritz MF, Dmochowska N, Badiee P, Cheah E, Kempson I, Berbeco RI, Thierry B. Formulation of simvastatin within high density lipoprotein enables potent tumour radiosensitisation. J Control Release. 2022;346:98–109. 35447296 10.1016/j.jconrel.2022.04.017

[87] Jing Z, Yuan W, Wang J, Ni R, Qin Y, Mao Z, Wei F, Song C, Zheng Y, Cai H, et al. Simvastatin/hydrogel-loaded 3D-printed titanium alloy scaffolds suppress osteosarcoma via TF/NOX2-associated ferroptosis while repairing bone defects. Bioact Mater. 2024;33:223–241. 38045570 10.1016/j.bioactmat.2023.11.001 PMC10689208

[88] Zambanini T, Borges R, De Souza ACS, Justo GZ, Machado J Jr, De Araujo DR, Marchi J. Holmium-containing bioactive glasses dispersed in poloxamer 407 hydrogel as a theragenerative composite for bone cancer treatment. Materials. 2021;14(6):1459. 33802678 10.3390/ma14061459 PMC8002559

[89] Stepanova M, Dobrodumov A, Averianov I, Gofman I, Nashchekina J, Guryanov I, Klyukin I, Zhdanov A, Korzhikova-Vlakh E, Zhizhin K. Design, fabrication and characterization of biodegradable composites containing closo-borates as potential materials for boron neutron capture therapy. Polymers. 2022;14(18):1459. 36146012 10.3390/polym14183864 PMC9506383

[90] Ding XL, Liu MD, Cheng Q, Guo WH, Niu MT, Huang QX, Zeng X, Zhang XZ. Multifunctional liquid metal-based nanoparticles with glycolysis and mitochondrial metabolism inhibition for tumor photothermal therapy. Biomaterials. 2022;281:121369. 35026671 10.1016/j.biomaterials.2022.121369

[91] Tang X, Tan L, Shi K, Peng J, Xiao Y, Li W, Chen L, Yang Q, Qian Z. Gold nanorods together with HSP inhibitor-VER-155008 micelles for colon cancer mild-temperature photothermal therapy. Acta Pharm Sin B. 2018;8(4):587–601. 30109183 10.1016/j.apsb.2018.05.011 PMC6089863

[92] Haixia X, Peng Z, Jiezhao L, Huiling G, Xie C, Yihan W, Yanglei J, Li J, Wang C, Wenning X, et al. 3D-printed magnesium peroxide-incorporated scaffolds with sustained oxygen release and enhanced photothermal performance for osteosarcoma multimodal treatments. ACS Appl Mater Interfaces. 2024;16(8):9626–9639. 38372238 10.1021/acsami.3c10807

[93] Paul S, Ghosh S, Kumar S. Tumor glycolysis, an essential sweet tooth of tumor cells. Semin Cancer Biol. 2022;86(Pt 3):1216–1230. 36330953 10.1016/j.semcancer.2022.09.007

[94] Zhang G, Cheng W, Du L, Xu C, Li J. Synergy of hypoxia relief and heat shock protein inhibition for phototherapy enhancement. J Nanobiotechnology. 2021;19(1):9. 33407570 10.1186/s12951-020-00749-5 PMC7789325

[95] Yu K, Zhou H, Xu Y, Cao Y, Zheng Y, Liang B. Engineering a triple-functional magnetic gel driving mutually-synergistic mild hyperthermia-starvation therapy for osteosarcoma treatment and augmented bone regeneration. J Nanobiotechnol. 2023;21(1):201. 10.1186/s12951-023-01955-7 PMC10291780 37365598

[96] Li Y, Zhang Y, Dong Y, Akakuru OU, Yao X, Yi J, Li X, Wang L, Lou X, Zhu B, et al. Ablation of gap junction protein improves the efficiency of nanozyme-mediated catalytic/starvation/mild-temperature photothermal therapy. Adv Mater. 2023;35(22): Article e2210464. 36964940 10.1002/adma.202210464

[97] Yan Z, Wu X, Tan W, Yan J, Zhou J, Chen S, Miao J, Cheng J, Shuai C, Deng Y. Single-atom Cu nanozyme-loaded bone scaffolds for ferroptosis-synergized mild photothermal therapy in osteosarcoma treatment. Adv Healthc Mater. 2024;13(15): Article e2304595. 38424663 10.1002/adhm.202304595

[98] He C, Yu L, Ding L, Yao H, Chen Y, Hao Y. Lysine demethylase KDM3A regulates nanophotonic hyperthermia resistance generated by 2D silicene in breast cancer. Biomaterials. 2020;255:120181. 32569864 10.1016/j.biomaterials.2020.120181

[99] Liang Y, Wang C, Yu S, Fan Y, Jiang Y, Zhou R, Yan W, Sun Y. IOX1 epigenetically enhanced photothermal therapy of 3D-printing silicene scaffolds against osteosarcoma with favorable bone regeneration. Mater Today Bio. 2023;23:100887. 10.1016/j.mtbio.2023.100887 PMC10746365 38144518

[100] Niu BY, Liao KX, Zhou YX, Wen T, Quan GL, Pan X, Wu CB. Application of glutathione depletion in cancer therapy: Enhanced ROS-based therapy, ferroptosis, and chemotherapy. Biomaterials. 2021;277:121110. 34482088 10.1016/j.biomaterials.2021.121110

[101] Chen WT, Liu JX, Zheng CY, Bai Q, Gao Q, Zhang YN, Dong K, Lu TL. Research progress on improving the efficiency of CDT by exacerbating tumor acidification. Int J Nanomedicine. 2022;17:2611–2628. 35712639 10.2147/IJN.S366187 PMC9196673

[102] Liu X, Zhang P, Xu M, Zhao Z, Yin X, Pu X, Wang J, Liao X, Huang Z, Cao S, et al. Mixed-valence vanadium-doped mesoporous bioactive glass for treatment of tumor-associated bone defects. J Mater Chem B. 2025;13(9):3138–3160. 39905825 10.1039/d4tb02290d

[103] Bai KK, Hong BH, He JL, Hong Z, Tan R. Preparation and antioxidant properties of selenium nanoparticles-loaded chitosan microspheres. Int J Nanomedicine. 2017;12:4527–4539. 28684913 10.2147/IJN.S129958 PMC5485894

[104] Hu T, Gu Z, Williams GR, Strimaite M, Zha J, Zhou Z, Zhang X, Tan C, Liang R. Layered double hydroxide-based nanomaterials for biomedical applications. Chem Soc Rev. 2022;51(14):6126–6176. 35792076 10.1039/d2cs00236a

[105] Bian Y, Zhao K, Hu T, Tan C, Liang R, Weng X. A Se nanoparticle/MgFe-LDH composite nanosheet as a multifunctional platform for osteosarcoma eradication, antibacterial and bone reconstruction. Adv Sci. 2024;11(33): Article e2403791. 10.1002/advs.202403791 PMC11434235 38958509

[106] Zheng X, Song X, Zhu G, Pan D, Li H, Hu J, Xiao K, Gong Q, Gu Z, Luo K, et al. Nanomedicine combats drug resistance in lung cancer. Adv Mater. 2024;36(3): Article e2308977. 37968865 10.1002/adma.202308977

[107] Xu R, Wang S, Guo Q, Zhong R, Chen X, Xia X. Anti-tumor strategies of photothermal therapy combined with other therapies using nanoplatforms. Pharmaceutics. 2025;17(3): Article e2308977. 10.3390/pharmaceutics17030306 PMC11944535 40142970

[108] Long X, Wang J, Wang H, Hu K, Zhang W, Lin W, Fang C, Cheng K, Song Z. Injectable 2D-MoS_2_-integrated bioadhesive hydrogel as photothermal-derived and drug-delivery implant for colorectal cancer therapy. Adv Healthc Mater. 2025;14(11): Article e2404842. 40091342 10.1002/adhm.202404842 PMC12023830

[109] Li S, Qing Y, Lou Y, Li R, Wang H, Wang X, Ying B, Tang X, Qin Y. Injectable thermosensitive black phosphorus nanosheet- and doxorubicin-loaded hydrogel for synergistic bone tumor photothermal-chemotherapy and osteogenesis enhancement. Int J Biol Macromol. 2023;239:124209. 36972826 10.1016/j.ijbiomac.2023.124209

[110] Liu X, Zhang Y, Wu H, Tang J, Zhou J, Zhao J, Wang S. A conductive gelatin methacrylamide hydrogel for synergistic therapy of osteosarcoma and potential bone regeneration. Int J Biol Macromol. 2023;228:111–122. 36563819 10.1016/j.ijbiomac.2022.12.185

[111] Chen S, Wang Y, Zhang X, Ma J, Wang M. Double-crosslinked bifunctional hydrogels with encapsulated anti-cancer drug for bone tumor cell ablation and bone tissue regeneration. Colloids Surf B Biointerfaces. 2022;213:112364. 35219965 10.1016/j.colsurfb.2022.112364

[112] Yao J, He Q, Zheng X, Shen S, Hui J, Fan D. An injectable hydrogel system with mild photothermal effects combined with ion release for osteosarcoma-related bone defect repair. Adv Funct Mater. 2024;34(30):2315217.

[113] Zhu C, He M, Sun D, Huang Y, Huang L, Du M, Wang J, Wang J, Li Z, Hu B, et al. 3D-printed multifunctional polyetheretherketone bone scaffold for multimodal treatment of osteosarcoma and osteomyelitis. ACS Appl Mater Interfaces. 2021;13(40):47327–47340. 34587454 10.1021/acsami.1c10898

[114] Rezk AI, Lee J, Kim BS, Chun S. Strategically designed bifunctional polydopamine enwrapping polycaprolactone-hydroxyapatite-doxorubicin composite nanofibers for osteosarcoma treatment and bone regeneration. ACS Appl Mater Interfaces. 2024;16(18):22946–22957. 38669442 10.1021/acsami.4c03015

[115] Wang C, Ye X, Zhao Y, Bai L, He Z, Tong Q, Xie X, Zhu H, Cai D, Zhou Y, et al. Cryogenic 3D printing of porous scaffolds for in situ delivery of 2D black phosphorus nanosheets, doxorubicin hydrochloride and osteogenic peptide for treating tumor resection-induced bone defects. Biofabrication. 2020;12(3):035004. 31952065 10.1088/1758-5090/ab6d35

[116] Wang L, Dai Z, Bi J, Chen Y, Wang Z, Sun Z, Ji Z, Wang H, Zhang Y, Wang L, et al. Polydopamine-functionalized calcium-deficient hydroxyapatite 3D-printed scaffold with sustained doxorubicin release for synergistic chemo-photothermal therapy of osteosarcoma and accelerated bone regeneration. Materials Today Bio. 2024;29:101253. 10.1016/j.mtbio.2024.101253 PMC11470592 39399244

[117] Rezk AI, Oh J-M, Abdal-Hay A, Lee J, Chun S, Kim B-S. Synergistic chemo-photothermal therapy and osteogenic activity using graphene oxide-functionalized composite whitlockite bone particles. J Mater Chem B. 2025;13(32):9838–9849. 40600257 10.1039/d5tb00240k

[118] Yan Z, Deng Y, Huang L, Zeng J, Wang D, Tong Z, Fan Q, Tan W, Yan J, Zang X, et al. Biopolymer-based bone scaffold for controlled Pt (IV) prodrug release and synergistic photothermal-chemotherapy and immunotherapy in osteosarcoma. J Nanobiotechnol. 2025;23(1):286. 10.1186/s12951-025-03253-w PMC11983740 40205459

[119] Hayakawa S, Ohishi T, Miyoshi N, Oishi Y, Nakamura Y, Isemura M. Anti-cancer effects of green tea epigallocatchin-3-gallate and coffee chlorogenic acid. Molecules. 2020;25(19):4553. 33027981 10.3390/molecules25194553 PMC7582793

[120] Liu Q, Zhang S, Shi L, Shi J, Sun C, Wang J, Zhou W, Zhou H, Shan F, Wang H, et al. Osteogenic induction and anti-inflammatory effects of calcium-chlorogenic acid nanoparticles remodel the osteoimmunology microenvironment for accelerating bone repair. Adv Healthc Mater. 2024;13(29): Article e2401114. 38885954 10.1002/adhm.202401114

[121] Yang YY, Zheng Y, Liu JJ, Chang ZP, Wang YH, Shao YY, Hou RG, Zhang X. Natural chlorogenic acid planted nanohybrids with steerable hyperthermia for osteosarcoma suppression and bone regeneration. Adv Healthc Mater. 2023;12(23): Article e2300325. 37167574 10.1002/adhm.202300325

[122] Deng TT, Ding WY, Lu XX, Zhang QH, Du JX, Wang LJ, Yang MN, Yin Y, Liu FJ. Pharmacological and mechanistic aspects of quercetin in osteoporosis. Front Pharmacol. 2024;15:1338951. 38333006 10.3389/fphar.2024.1338951 PMC10851760

[123] Zhang G, Wu Z, Yang Y, Shi J, Lv J, Fang Y, Shen Z, Lv Z, Li P, Yao X, et al. A multifunctional antibacterial coating on bone implants for osteosarcoma therapy and enhanced osteointegration. Chem Eng J. 2022;428:131155.

[124] Xu B, Li S, Shi R, Liu H. Multifunctional mesoporous silica nanoparticles for biomedical applications. Signal Transduct Target Ther. 2023;8(1):435. 37996406 10.1038/s41392-023-01654-7 PMC10667354

[125] Shi Q, Lu Y, Zhang G, Yang X, Li R, Zhang G, Guo X, Song J, Ding Q. Multifunctional mesoporous silica nanoparticles for pH-response and photothermy enhanced osteosarcoma therapy. Colloids Surf B Biointerfaces. 2022;217:112615. 35759893 10.1016/j.colsurfb.2022.112615

[126] Xu C, Wang M, Guo W, Sun W, Liu Y. Curcumin in osteosarcoma therapy: Combining with immunotherapy, chemotherapeutics, bone tissue engineering materials and potential synergism with photodynamic therapy. Front Oncol. 2021;11:672490. 34094974 10.3389/fonc.2021.672490 PMC8172965

[127] Astaneh ME, Noori F, Fereydouni N. Curcumin-loaded scaffolds in bone regeneration. Heliyon. 2024;10(11): Article e32566. 38961905 10.1016/j.heliyon.2024.e32566 PMC11219509

[128] Tan B, Wu Y, Wu Y, Shi K, Han R, Li Y, Qian Z, Liao J. Curcumin-microsphere/IR820 hybrid bifunctional hydrogels for in situ osteosarcoma chemo-co-thermal therapy and bone reconstruction. ACS Appl Mater Interfaces. 2021;13(27):31542–31553. 34191477 10.1021/acsami.1c08775

[129] Han R, Min Y, Li G, Chen S, Xie M, Zhao Z. Supercritical CO_2_-assisted fabrication of CM-PDA/SF/nHA nanofibrous scaffolds for bone regeneration and chemo-photothermal therapy against osteosarcoma. Biomater Sci. 2023;11(15):5218–5231. 37338001 10.1039/d3bm00532a

[130] Chauhan A, Saini A, Sharma D. The evolution of integrated magnetic hyperthermia and chemodynamic therapy for combating cancer: A comprehensive viewpoint. Nanoscale Adv. 2025;7(16):4820–4836. 40708892 10.1039/d4na01004c PMC12285877

[131] Zhuang H, Qin C, Zhang M, Ma J, Zhai D, Ma B, Ma N, Huan Z, Wu C. 3D-printed bioceramic scaffolds with Fe_3_S_4_ microflowers for magnetothermal and chemodynamic therapy of bone tumor and regeneration of bone defects. Biofabrication. 2021;13(4): 10.1088/1758-5090/ac19c7. 34340226

[132] Xiang H, Feng W, Chen Y. Single-atom catalysts in catalytic biomedicine. Adv Mater. 2020;32(8): Article e1905994. 31930751 10.1002/adma.201905994

[133] Jiao L, Yan HY, Wu Y, Gu WL, Zhu CZ, Du D, Lin YH. When nanozymes meet single-atom catalysis. Angew Chem Int Ed Engl. 2020;59(7):2565–2576. 31209985 10.1002/anie.201905645

[134] Wang L, Yang Q, Huo M, Lu D, Gao Y, Chen Y, Xu H. Engineering single-atomic iron-catalyst-integrated 3D-printed bioscaffolds for osteosarcoma destruction with antibacterial and bone defect regeneration bioactivity. Adv Mater. 2021;33(31): Article e2100150. 34146359 10.1002/adma.202100150

[135] Kahlson MA, Dixon SJ. Copper-induced cell death. Science. 2022;375(6586):1231–1232. 35298241 10.1126/science.abo3959

[136] Kim B, Brueggemeyer MT, Transue WJ, Park Y, Cho J, Siegler MA, Solomon EI, Karlin KD. Fenton-like chemistry by a copper(I) complex and H_2_O_2_ relevant to enzyme peroxygenase C-H hydroxylation. J Am Chem Soc. 2023;145(21):11735–11744. 37195014 10.1021/jacs.3c02273 PMC10364799

[137] Huang B, Li G, Cao L, Wu S, Zhang Y, Li Z, Zhou F, Xu K, Wang G, Su J. Nanoengineered 3D-printing scaffolds prepared by metal-coordination self-assembly for hyperthermia-catalytic osteosarcoma therapy and bone regeneration. J Colloid Interface Sci. 2024;672:724–735. 38870763 10.1016/j.jcis.2024.06.055

[138] Ye L, Yu C, Xia J, Ni K, Zhang Y, Ying X, Xie D, Jin Y, Sun R, Tang R, et al. Multifunctional nanomaterials via cell cuproptosis and oxidative stress for treating osteosarcoma and OS-induced bone destruction. Mater Today Bio. 2024;25:100996. 10.1016/j.mtbio.2024.100996 PMC10900125 38420143

[139] Yan S, Zhang F, Luo L, Wang L, Liu Y, Leng J. Shape memory polymer composites: 4D printing, smart structures, and applications. Research. 2023;6:0234. 37941913 10.34133/research.0234 PMC10629366

[140] Overchuk M, Weersink RA, Wilson BC, Zheng G. Photodynamic and photothermal therapies: Synergy opportunities for nanomedicine. ACS Nano. 2023;17(9):7979–8003. 37129253 10.1021/acsnano.3c00891 PMC10173698

[141] Ma Q, Xu S, Wang Q, Que Y, He P, Yang R, Wang H, Wu Z, Xiao L, Yuan X, et al. Controllable all-in-one biomimetic hollow nanoscaffold initiating pyroptosis-mediated antiosteosarcoma targeted therapy and bone defect repair. ACS Appl Mater Inter. 2024;16(49):67424–67443. 10.1021/acsami.4c16287 PMC11647757 39603818

[142] Yang C, Tan QY, Li Q, Zhou J, Fan JJ, Li B, Sun J, Lv KL. 2D/2D TiCMXene/g-CN nanosheets heterojunction for high efficient CO reduction photocatalyst: Dual effects of urea. Appl Catal B Environ. 2020;268:118738.

[143] Zhang G, Lu Y, Song J, Huang D, An M, Chen W, Han P, Yao X, Zhang X. A multifunctional nano-hydroxyapatite/MXene scaffold for the photothermal/dynamic treatment of bone tumours and simultaneous tissue regeneration. J Colloid Interface Sci. 2023;652(Pt B):1673–1684. 37666199 10.1016/j.jcis.2023.08.176

[144] Gong C, Wang J, Tang F, Tong D, Wang Z, Zhou Z, Ruan R, Zhang J, Song J, Yang H. Bionic bilayer scaffold for synchronous hyperthermia therapy of orthotopic osteosarcoma and osteochondral regeneration. ACS Appl Mater Interfaces. 2024;16(7):8538–8553. 38343191 10.1021/acsami.3c18171

[145] Liao J, Shi K, Jia Y, Wu Y, Qian Z. Gold nanorods and nanohydroxyapatite hybrid hydrogel for preventing bone tumor recurrence via postoperative photothermal therapy and bone regeneration promotion. Bioact Mater. 2021;6(8):2221–2230. 33553811 10.1016/j.bioactmat.2021.01.006 PMC7829101

[146] Ma L, Zhou J, Wu Q, Luo G, Zhao M, Zhong G, Zheng Y, Meng X, Cheng S, Zhang Y. Multifunctional 3D-printed scaffolds eradiate orthotopic osteosarcoma and promote osteogenesis via microwave thermo-chemotherapy combined with immunotherapy. Biomaterials. 2023;301:122236. 37506512 10.1016/j.biomaterials.2023.122236

[147] Soundararajan L, Dharmarajan A, Samji P. Regulation of pleiotropic physiological roles of nitric oxide signaling. Cell Signal. 2023;101:110496. 36252791 10.1016/j.cellsig.2022.110496

[148] Zhang Y, Mou H, Huang Q, Jian CC, Li XL, Chen SA, Chen YX, Tao BL, Ou YS. Engineering of phytic acid-coated Prussian nanoparticles for combined nitric oxide and low-temperature photothermal therapy of osteosarcoma. Chem Eng J. 2024;491:151730.

[149] Yang Q, Yin H, Xu T, Zhu D, Yin J, Chen Y, Yu X, Gao J, Zhang C, Chen Y, et al. Engineering 2D mesoporous silica@MXene-integrated 3D-printing scaffolds for combinatory osteosarcoma therapy and NO-augmented bone regeneration. Small. 2020;16(14): Article e1906814. 32108432 10.1002/smll.201906814

[150] Lu Y, Liu A, Jin S, Dai J, Yu Y, Wen P, Zheng Y, Xia D. Additively manufactured biodegradable Zn-based porous scaffolds to suppress osteosarcoma and promote osteogenesis. Adv Mater. 2024;37(3): Article e2410589. 39564691 10.1002/adma.202410589

[151] Zhou J, Lin G, Fu X, Qiu S, Zhang Y, Chen X, Liu Y, Wan X, Li Z, Li Y, et al. ZIF-8-modified multifunctional hydrogel loading siRNA and DOX for postoperative therapy of maxillofacial osteosarcoma and bone repair. ACS Appl Mater Interfaces. 2025;17(12):17990–18002. 40079695 10.1021/acsami.4c21331

[152] Matsuoka K, Bakiri L, Wolff LI, Linder M, Mikels-Vigdal A, Patino-Garcia A, Lecanda F, Hartmann C, Sibilia M, Wagner EF. Wnt signaling and Loxl2 promote aggressive osteosarcoma. Cell Res. 2020;30(10):885–901. 32686768 10.1038/s41422-020-0370-1 PMC7608146

[153] Yao Z, Han L, Chen Y, He F, Sun B, Kamar S, Zhang Y, Yang Y, Wang C, Yang Z. Hedgehog signalling in the tumourigenesis and metastasis of osteosarcoma, and its potential value in the clinical therapy of osteosarcoma. Cell Death Dis. 2018;9(6):701. 29899399 10.1038/s41419-018-0647-1 PMC5999604

[154] Yuan J, Jia J, Wu T, Du Z, Chen Q, Zhang J, Wu Z, Yuan Z, Zhao X, Liu J, et al. Long intergenic non-coding RNA DIO3OS promotes osteosarcoma metastasis via activation of the TGF-beta signaling pathway: A potential diagnostic and immunotherapeutic target for osteosarcoma. Cancer Cell Int. 2023;23(1):215. 37752544 10.1186/s12935-023-03076-5 PMC10521498

[155] Cascio S, Chandler C, Zhang L, Sinno S, Gao B, Onkar S, Bruno TC, Vignali DA, Mahdi H, Osmanbeyoglu HU, et al. Cancer-associated MSC drive tumor immune exclusion and resistance to immunotherapy, which can be overcome by hedgehog inhibition. Sci Adv. 2021;7(46):eabi5790. 34767446 10.1126/sciadv.abi5790 PMC8589308

[156] Chu X, Mi B, Xiong Y, Wang R, Liu T, Hu L, Yan C, Zeng R, Lin J, Fu H, et al. Bioactive nanocomposite hydrogel enhances postoperative immunotherapy and bone reconstruction for osteosarcoma treatment. Biomaterials. 2024;312:122714. 39079462 10.1016/j.biomaterials.2024.122714

[157] Xie D, Hu C, Zhu Y, Yao J, Li J, Xia J, Ye L, Jin Y, Jiang S, Hu T, et al. Sequential therapy for osteosarcoma and bone regeneration via chemodynamic effect and cuproptosis using a 3D-printed scaffold with TME-responsive hydrogel. Small. 2024;21(5): Article e2406639. 39908123 10.1002/smll.202406639

[158] Li C, Zhang W, Nie Y, Du X, Huang C, Li L, Long J, Wang X, Tong W, Qin L, et al. Time-sequential and multi-functional 3D printed MgO(2)/PLGA scaffold developed as a novel biodegradable and bioactive bone substitute for challenging postsurgical osteosarcoma treatment. Adv Mater. 2024;36(34): Article e2308875. 38091500 10.1002/adma.202308875

[159] Zhang W, Li L, Wang Z, Nie Y, Yang Y, Li C, Zhang Y, Jiang Y, Kou Y, Zhang W, et al. Injectable and adhesive MgO2-potentiated hydrogel with sequential tumor synergistic therapy and osteogenesis for challenging postsurgical osteosarcoma treatment. Biomaterials. 2025;315:122959. 39612764 10.1016/j.biomaterials.2024.122959

[160] Yang XL, Ju XJ, Mu XT, Wang W, Xie R, Liu Z, Chu LY. Core-shell chitosan microcapsules for programmed sequential drug release. ACS Appl Mater Interfaces. 2016;8(16):10524–10534. 27052812 10.1021/acsami.6b01277

[161] Singh B, Kim J, Shukla N, Lee J, Kim K, Park MH. Smart delivery platform using core-shell nanofibers for sequential drug release in wound healing. ACS Appl Bio Mater. 2023;6(6):2314–2324. 10.1021/acsabm.3c00178 37254937

[162] Luan X, Zhang X, Luan Q, Gan J, Wang Y, Zhao Y. Traditional Chinese medicine integrated multifunctional responsive core–shell microneedles for dermatosis treatment. Research. 2024;7:0420. 38966748 10.34133/research.0420 PMC11223756

[163] Zhang WL, Dai ZW, Chen SY, Guo WX, Wang ZW, Wei JS. A novel poly(3-hydroxybutyrate-co-3-hydroxyvalerate) (PHBV)-PEG-melatonin composite scaffold enhances for inhibiting bone tumor recurrence and enhancing bone regeneration. Front Pharmacol. 2023;14:1246783. 37663244 10.3389/fphar.2023.1246783 PMC10469957

[164] Al-Ansari N, Samuel SM, Busselberg D. Unveiling the protective role of melatonin in osteosarcoma: Current knowledge and limitations. Biomolecules. 2024;14(2):145. 38397382 10.3390/biom14020145 PMC10886489

[165] Huang W, Wu X, Zhao Y, Liu Y, Zhang B, Qiao M, Zhu Z, Zhao Z. Janus-inspired core-shell structure hydrogel programmatically releases melatonin for reconstruction of postoperative bone tumor. ACS Appl Mater Interfaces. 2023;15(2):2639–2655. 36603840 10.1021/acsami.2c18545 PMC9869893

[166] Liu K, Li L, Li Y, Luo Y, Zhang Z, Wen W, Ding S, Huang Y, Liu M, Zhou C, et al. Creating a bionic scaffold via light-curing liquid crystal ink to reveal the role of osteoid-like microenvironment in osteogenesis. Bioact Mater. 2024;40:244–260. 38973990 10.1016/j.bioactmat.2024.06.019 PMC11226751

[167] Li L, Lin Y, Liu K, Huang R, Wen W, Huang Y, Liu M, Zhou C, Ding S, Luo B. Multiple-effect combined hydrogels: “Temporal regulation” treatment of osteosarcoma-associated bone defects with switchable hyperthermia and bioactive agents. Adv Healthc Mater. 2024;13(31): Article e2402505. 39233538 10.1002/adhm.202402505

[168] Guo Z, Xie W, Lu J, Guo X, Chi Y, Wang D, Takuya N, Xu W, Ye J, Liu X, et al. Ferrous ions doped layered double hydroxide: Smart 2D nanotheranostic platform with imaging-guided synergistic chemo/photothermal therapy for breast cancer. Biomater Sci. 2021;9(17):5928–5938. 34308465 10.1039/d1bm00765c

[169] Zhang X, Cheng G, Xing X, Liu J, Cheng Y, Ye T, Wang Q, Xiao X, Li Z, Deng H. Near-infrared light-triggered porous AuPd alloy nanoparticles to produce mild localized heat to accelerate bone regeneration. J Phys Chem Lett. 2019;10(15):4185–4191. 31295998 10.1021/acs.jpclett.9b01735

[170] Yu Z, Wang H, Ying B, Mei X, Zeng D, Liu S, Qu W, Pan X, Pu S, Li R, et al. Mild photothermal therapy assist in promoting bone repair: Related mechanism and materials. Mater Today Bio. 2023;23:100834. 10.1016/j.mtbio.2023.100834 PMC10643361 38024841

[171] Bai J, Zhang Y, Tang C, Hou Y, Ai X, Chen X, Zhang Y, Wang X, Meng X. Gallic acid: Pharmacological activities and molecular mechanisms involved in inflammation-related diseases. Biomed Pharmacother. 2021;133:110985. 33212373 10.1016/j.biopha.2020.110985

[172] Jiang Y, Pei J, Zheng Y, Miao YJ, Duan BZ, Huang LF. Gallic acid: A potential anti-cancer agent. Chin J Integr Med. 2022;28(7):661–671. 34755289 10.1007/s11655-021-3345-2

[173] Hou X, Zhang L, Chen Y, Liu Z, Zhao X, Lu B, Luo Y, Qu X, Musskaya O, Glazov I, et al. Photothermal switch by gallic acid-calcium grafts synthesized by coordination chemistry for sequential treatment of bone tumor and regeneration. Biomaterials. 2024;312:122724. 39106818 10.1016/j.biomaterials.2024.122724

[174] Wang X, Dai X, Chen Y. Sonopiezoelectric nanomedicine and materdicine. Small. 2023;19(29): Article e2301693. 37093550 10.1002/smll.202301693

[175] Liu Z, Wan X, Wang ZL, Li L. Electroactive biomaterials and systems for cell fate determination and tissue regeneration: Design and applications. Adv Mater. 2021;33(32): Article e2007429. 34117803 10.1002/adma.202007429

[176] Khare D, Basu B, Dubey AK. Electrical stimulation and piezoelectric biomaterials for bone tissue engineering applications. Biomaterials. 2020;258:120280. 32810650 10.1016/j.biomaterials.2020.120280

[177] Yang S, Wang Y, Liang X. Piezoelectric nanomaterials activated by ultrasound in disease treatment. Pharmaceutics. 2023;15(5):1338. 37242580 10.3390/pharmaceutics15051338 PMC10223188

[178] Xu Y, Xu C, Song H, Feng X, Ma L, Zhang X, Li G, Mu C, Tan L, Zhang Z, et al. Biomimetic bone-periosteum scaffold for spatiotemporal regulated innervated bone regeneration and therapy of osteosarcoma. J Nanobiotechnology. 2024;22(1):250. 38750519 10.1186/s12951-024-02430-7 PMC11094931

[179] Zhang C, Lei D, Xie C, Hang X, He C, Jiang HL. Piezo-photocatalysis over metal-organic frameworks: Promoting photocatalytic activity by piezoelectric effect. Adv Mater. 2021;33(51): Article e2106308. 34642997 10.1002/adma.202106308

[180] Liu W, Wang P, Ao Y, Chen J, Gao X, Jia B, Ma T. Directing charge transfer in a chemical-bonded BaTiO_3_ @ReS_2_ Schottky heterojunction for piezoelectric enhanced photocatalysis. Adv Mater. 2022;34(29): Article e2202508. 35560713 10.1002/adma.202202508

[181] Xiao C, Wang R, Fu R, Yu P, Guo J, Li G, Wang Z, Wang H, Nie J, Liu W, et al. Piezo-enhanced near infrared photocatalytic nanoheterojunction integrated injectable biopolymer hydrogel for anti-osteosarcoma and osteogenesis combination therapy. Bioact Mater. 2024;34:381–400. 38269309 10.1016/j.bioactmat.2024.01.003 PMC10806218

[182] Rong X, Xiao S, Geng W, Zhu B, Mou P, Ding Z, Zhang B, Fan Y, Qiu L, Cheng C. Sono-activable and biocatalytic 3D-printed scaffolds for intelligently sequential therapies in osteosarcoma eradication and defect regeneration. Nat Commun. 2025;16(1):6150. 40610512 10.1038/s41467-025-61377-x PMC12229518

[183] Wang X, Guo X, Ren H, Song X, Chen L, Yu L, Ren J, Chen Y. An “outer piezoelectric and inner epigenetic” logic-gated PANoptosis for osteosarcoma sono-immunotherapy and bone regeneration. Adv Mater. 2024;37(7): Article e2415814. 39726343 10.1002/adma.202415814

[184] Lang ST, Gan LS, Mclennan C, Monchi O, Kelly JJP. Impact of peritumoral edema during tumor treatment field therapy: A computational modelling study. IEEE Trans Biomed Eng. 2020;67(12):3327–3338. 32286953 10.1109/TBME.2020.2983653

[185] Xiao C, Fan L, Zhou S, Kang X, Guan P, Fu R, Li C, Ren J, Wang Z, Yu P, et al. One-dimensional ferroelectric nanoarrays with wireless switchable static and dynamic electrical stimulation for selective regulating osteogenesis and antiosteosarcoma. ACS Nano. 2022;16(12):20770–20785. 36412574 10.1021/acsnano.2c07900

[186] Huzum B, Puha B, Necoara R, Gheorghevici S, Puha G, Filip A, Sirbu P, Alexa O. Biocompatibility assessment of biomaterials used in orthopedic devices: An overview (review). Exp Ther Med. 2021;22(5):1315. 34630669 10.3892/etm.2021.10750 PMC8461597

[187] Miwa S, Yamamoto N, Hayashi K, Takeuchi A, Igarashi K, Tsuchiya H. Surgical site infection after bone tumor surgery: Risk factors and new preventive techniques. Cancer. 2022;14(18):4527. 10.3390/cancers14184527 PMC9497226 36139686

[188] Rosa V, Silikas N, Yu B, Dubey N, Sriram G, Zinelis S, Lima AF, Bottino MC, Ferreira JN, Schmalz G, et al. Guidance on the assessment of biocompatibility of biomaterials: Fundamentals and testing considerations. Dent Mater. 2024;40(11):1773–1785. 39129079 10.1016/j.dental.2024.07.020

[189] Luo Y, Zhang H, Wang Z, Jiao J, Wang Y, Jiang W, Yu T, Liu H, Guan L, Li M, et al. Strategic incorporation of metal ions in bone regenerative scaffolds: Multifunctional platforms for advancing osteogenesis. Regen Biomater. 2025;12:rbaf068. 40755870 10.1093/rb/rbaf068 PMC12317318

[190] Farjaminejad S, Farjaminejad R, Hasani M, Garcia-Godoy F, Abdouss M, Marya A, Harsoputranto A, Jamilian A. Advances and challenges in polymer-based scaffolds for bone tissue engineering: A path towards personalized regenerative medicine. Polymers. 2024;16(23):3303. 39684048 10.3390/polym16233303 PMC11644794

[191] Cortini M, Armirotti A, Columbaro M, Longo DL, Di Pompo G, Cannas E, Maresca A, Errani C, Longhi A, Righi A, et al. Exploring metabolic adaptations to the acidic microenvironment of osteosarcoma cells unveils sphingosine 1-phosphate as a valuable therapeutic target. Cancer. 2021;13(2):311. 10.3390/cancers13020311 PMC7830496 33467731

[192] Balcucho J, Narváez DM, Castro-Mayorga JL. Antimicrobial and biocompatible polycaprolactone and copper oxide nanoparticle wound dressings against methicillin-resistant *Staphylococcus aureus*. Nano. 2020;10(9):1692. 10.3390/nano10091692 PMC7560150 32872095

[193] Tipnis NP, Burgess DJ. Sterilization of implantable polymer-based medical devices: A review. Int J Pharm. 2018;544(2):455–460. 29274370 10.1016/j.ijpharm.2017.12.003

[194] Shen X, Zhang Z, Cheng C, Liu C, Ma N, Sun D, Li D, Wang C. Bone regeneration and antibacterial properties of calcium-phosphorus coatings induced by gentamicin-loaded polydopamine on magnesium alloys. Biomed Technol. 2024;5:87–101.

[195] Morizane R, Lamers MM. Organoids in disease modeling and regenerative medicine. Cell Mol Life Sci. 2025;82(1):169. 40257505 10.1007/s00018-025-05692-y PMC12011692

[196] Ucci A, Giacchi L, Rucci N. Primary bone tumors and breast cancer-induced bone metastases: In vivo animal models and new alternative approaches. Biomedicines. 2024;12(11):2451. 39595017 10.3390/biomedicines12112451 PMC11591690

[197] Zhang W, Li W, Yin C, Feng C, Liu B, Xu H, Jin X, Tu C, Li Z. PRKDC induces chemoresistance in osteosarcoma by recruiting GDE2 to stabilize GNAS and activate AKT. Cancer Res. 2024;84(17):2873–2887. 38900943 10.1158/0008-5472.CAN-24-0163 PMC11372366

[198] Zhang X, Zhao Z, Wang X, Zhang S, Zhao Z, Feng W, Xu L, Nie J, Li H, Liu J, et al. Deprivation of methionine inhibits osteosarcoma growth and metastasis via C1orf112-mediated regulation of mitochondrial functions. Cell Death Dis. 2024;15(5):349. 38769167 10.1038/s41419-024-06727-1 PMC11106329

